# A novel four-gene of iron metabolism-related and methylated for prognosis prediction of hepatocellular carcinoma

**DOI:** 10.1080/21655979.2020.1866303

**Published:** 2020-12-31

**Authors:** Huimin Shen, Hao Wu, Fengkai Sun, Jianni Qi, Qiang Zhu

**Affiliations:** a Department of Gastroenterology, Shandong Provincial Hospital, Cheeloo College of Medicine, Shandong University, Jinan, China; b Central Laboratory, Shandong Provincial Hospital Affiliated to Shandong First Medical University, Jinan, China

**Keywords:** Hepatocellular carcinoma, iron metabolism, DNA methylation, prognosis, TCGA, ICGC

## Abstract

Hepatocellular carcinoma (HCC) is a liver disease with a complex underlying mechanism, and patients with HCC have low survival rates. Iron metabolism plays a crucial role in the pathogenesis of HCC; however, the prognostic value of iron metabolism-related and methylated genes for HCC needs to be further explored. In the present study, we identified differentially expressed genes (DEGs) that play a role in iron metabolism and DNA methylation in HCC from The Cancer Genome Atlas. Four of these DEGs, whose expression levels are correlated with HCC prognosis, namely, RRM2, FTCD, CYP2C9, and ATP6V1C1, were further used to construct a prognostic model for HCC, wherein the risk score was calculated using the gene expression of the four DEGs. This could be used to predict the overall survival of HCC patients for 1, 3, and 5 years. Results of a multivariate Cox regression analysis further indicated that the risk score was an independent variable correlated with the prognosis of HCC patients. The identified gene signature was further validated using an independent cohort of HCC patients from the International Cancer Genome Consortium. Weighted gene co-expression network analysis and gene set enrichment analysis were performed to identify potential regulatory mechanisms of the gene signature in HCC. Taken together, we identified key prognostic factors of iron metabolism-related and methylated genes for HCC, providing a potential treatment strategy for HCC.

## Introduction

**Figure:**
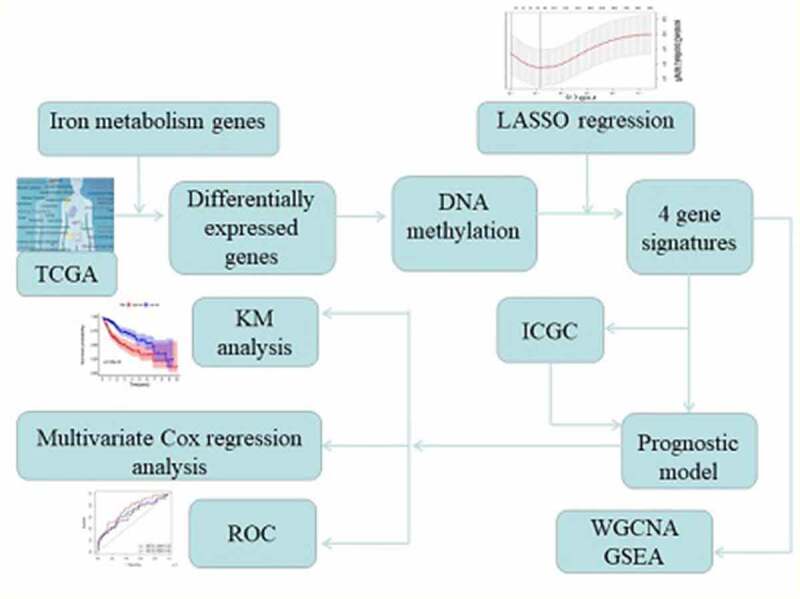

Liver cancer is one of the most common forms of malignancy worldwide, ranking sixth in terms of morbidity and fourth in terms of mortality [1]. Hepatocellular carcinoma (HCC) is the main type of primary liver cancer, accounting for 75–85% of all liver cancers [2]. Currently, major risk factors for HCC consist of the hepatitis B virus, the hepatitis C virus, alcohol addiction, exposure to aflatoxins, and nonalcoholic fatty liver [3]. Most HCC patients are diagnosed in the advanced stage and thus miss the optimal stage that is receptive to treatment. In addition, HCC is not sensitive to chemotherapy and radiotherapy. Despite advances in surgical techniques and therapeutic regimens, advanced HCC has extremely poor prognosis [4] and a high recurrence rate after surgical resection. Therefore, an effective prognostic model for HCC is an urgent concern.

Iron is an essential element for all living organisms, and it participates in a variety of important physiological processes. It is well established that iron both promotes tumor cell growth as well as cell death. Studies have reported an impaired iron metabolism in several tumors [5–7], contributing to tumor genesis, development, invasion, and metastasis [8]. An impaired iron metabolism is an important risk factor for HCC, since the liver is the main storage site for iron. Increasingly, studies have shown that hepatic iron overload is involved in the pathogenesis of HCC. However, whether iron metabolism plays a role in the prognosis of HCC is unknown.

The initiation and progression of HCC are regulated by both genetic and epigenetic alterations. DNA methylation, one of the central epigenetic modifications, has been extensively studied in cancer biology [9]. Studies have confirmed that hypomethylation of oncogenes and hypermethylation of tumor suppressor genes are the main forms of abnormal DNA methylation in cancer [10]. Importantly, Zhang et al. reported that the aberrant expression of iron metabolism genes in different kinds of cancers might be related to the regulation of DNA methylation [5]. However, the link between DNA methylation of iron metabolism-related genes and the pathogenesis of HCC remains unknown and deserves further investigation.

In this study, we identified four genes involved in iron metabolism and DNA methylation, whose expression levels are correlated with the prognosis of HCC, and used them to construct a prognostic model that can predict the prognosis of HCC, independent of other variables. Our results help predict the prognosis of HCC patients and provide a new direction for the development of personalized treatment strategies.

## Materials and methods

### Data acquisition

RNA sequencing (RNA-seq) data, DNA methylation data, and clinical information of 371 HCC patients were extracted from The Cancer Genome Atlas (TCGA) for subsequent analysis (**Supplementary Table 1**). The RNA-seq data and clinical information of additional 243 patients with HCC were obtained from the International Cancer Genome Consortium (ICGC) LIRI-JP cohort and was used for validation. Sixteen iron metabolism-related gene sets were obtained from the Molecular Signatures Database (MSigDB). A total of 514 genes were extracted from the iron metabolism-related gene sets after removing overlapping genes (**Supplementary Table 2**).

### Identification of prognostic factors for HCC

Differentially expressed genes (DEGs) involved in iron metabolism were identified between tumor tissues and normal tissues from TCGA (|log2FC| > 1 and *P*-value < 0.05) using the *Limma* package in *R*. A volcano plot of the DEGs involved in iron metabolism was constructed with the *ggplot2* package in *R*. Methylation of CpG sites in each gene was detected. To identify the genes that affect HCC prognosis, LASSO Cox regression model was constructed using the *glmnet* package in *R*.

### Construction of the prognostic model and validation using the patient cohort from ICGC

Univariate and multivariate Cox analyses for overall survival (OS) were performed to identify prognostic factors of HCC. We adjusted *P*-value using the Benjamini & Hochberg correction. The risk scores of HCC patients were calculated using the gene expression levels and corresponding coefficients in TCGA and the ICGC. The median risk score was used to classify patients into high- and low-risk groups. The Kaplan-Meier (KM) analysis was performed between the two risk groups with long-rank test. Additionally, time-dependent receiver operating characteristic (ROC) curve was constructed for the cohorts from TCGA and the ICGC using the *survivalROC* package in *R*. Using the multivariate Cox analysis, we determined if the calculated risk score could act as an independent prognosis factor for HCC using data from TCGA.

### Weighted gene co-expression network analysis (WGCNA)

WGCNA was used for the construction of a gene co-expression network, which is a representation of related gene expression profile patterns. We used the WGCNA package in *R* to assess the significance of gene signatures associated with the prognosis of HCC. The co-expression network of the gene signature, comprised of four DEGs, was visualized using Cytoscape.

### Gene set enrichment analysis (GSEA)

GSEA is a method designed by the Broad Institute Gene Set Enrichment Analysis for functional analysis of gene expression. We conducted GSEA to identify important Kyoto encyclopedia of genes and genomes (KEGG) pathways associated with the progression of HCC. The enrichment analysis of KEGG pathways was conducted in the high-risk group using GSEA tools.

### Clinical samples

We harvested cancerous and adjacent non-cancerous specimens from 10 HCC patients who underwent surgery in the Shandong Provincial Hospital from September to October 2020. Histopathological sections were formalin-fixed, paraffin-embedded, and classified into cancerous or non-cancerous specimens by two pathologists. Specimen collection protocols were approved by the Medical Ethics Committee of Shandong Provincial Hospital, and all patients provided written informed consent before tissue harvesting.

### Immunohistochemical (IHC) Staining

Paraffin-embedded samples were cut into 3-μm thick sections. After deparaffinization, rehydration, and antigen retrieval, the sections were incubated with 3% H_2_O_2_ at room temperature to block endogenous peroxidases, followed by incubation with 3% bovine serum albumin. Then, sections were incubated with primary rabbit polyclonal anti-RRM2 antibody (1:50, 11661-1-AP, Proteintech, China), primary rabbit polyclonal anti-FTCD antibody (1:50, 21959-1-AP, Proteintech, China), primary rabbit polyclonal anti-CYP2C9 antibody (1:200, 16546-1-AP, Proteintech, China), and primary rabbit polyclonal anti-ATP6V1C1 antibody (1:50, 16054-1-AP, Proteintech, China) at 4 °C overnight, followed by an incubation with the corresponding secondary antibodies at 37 °C for 30 minutes. 3ʹ3-diaminobenzidine was used as a chromogen for visualization, and sections were counterstained with hematoxylin, dehydrated, and mounted. Sections were observed with an E100 microscope (Nikon), acquisitive and analysis images.

### Statistical analysis

*R* was used for all statistical analyses. Univariate and multivariate Cox regression analyses were used to screen the independent factors for HCC prognosis. OS of the two risk groups was estimated from survival curves generated using KM analysis, and the log-rank test was used to compare the OS of both groups. *P*-value less than 0.05 was considered significant.

## Results

In the present study, we aimed to identify genes involved in iron metabolism and DNA methylation that affect the prognosis of HCC. We identified four DEGs involved in iron metabolism, whose expression and methylation levels are associated with the prognosis of HCC, and these DEGs were used to construct a novel prognostic model. In addition, we performed functional enrichment analysis to explore the potential metabolic and biosynthetic mechanisms that are involved in the pathogenesis of HCC.

### Four DEGs in HCC tissue were identified as prognostic factors for HCC

**Figure 1..**
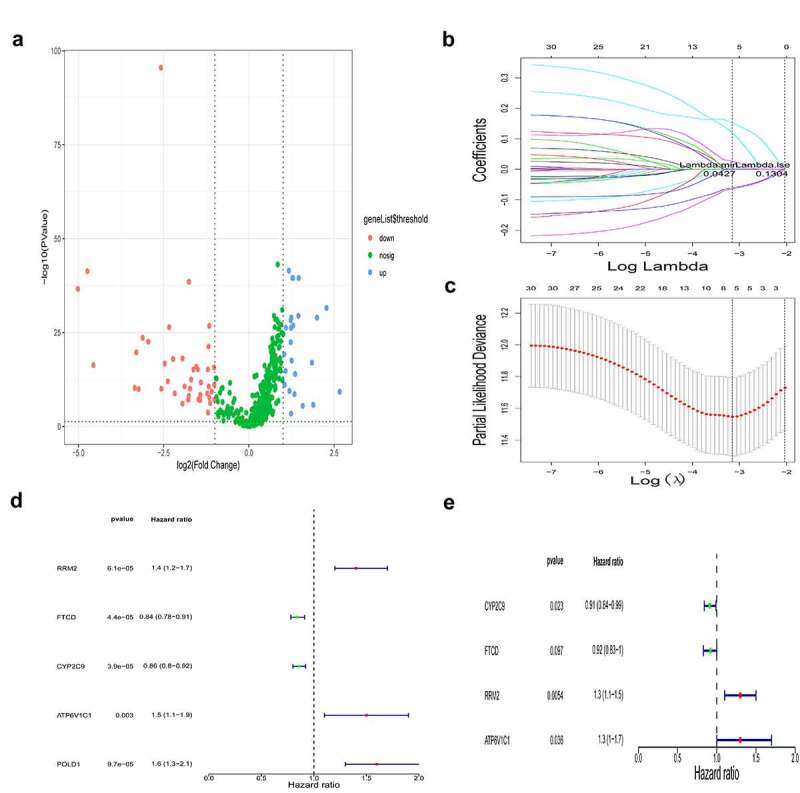
Identification of genes that act as prognostic factors for HCC

To identify genes involved in iron metabolism and DNA methylation that affect the prognosis of HCC, we firstly identified DEGs between cancerous and non-cancerous tissue using transcriptome data of HCC from TCGA (**Supplementary Table 3**). Gene sets related to iron metabolism from the MSigDB database were combined with the aforementioned DEGs to identify DEGs involved iron metabolism in HCC. We identified 71 DEGs related to iron metabolism between the cancerous and non-cancerous groups, of which 29 were upregulated and 42 were downregulated, and are presented in Figure 1a. We downloaded the 450 K methylation data of patients with HCC from TCGA, and performed a correlation analysis between the expression level and CpG site methylation of the 71 co-differentially expressed genes in HCC. The methylation status of the identified DEGs is listed in Supplementary Table 4.

(a) Volcano map describes the distribution of upregulated and downregulated differentially expressed genes (DEGs) involved in iron metabolism genes. Red, green and blue dots represent downregulated, no change and upregulated DEGs. (b-c) LASSO Cox regression analysis was used to identify prognostic factors for HCC from TCGA. (d) Univariate Cox regression analysis of prognostic factors. (e) Multivariate Cox regression analysis of prognostic factors.

To address which iron metabolism-related and methylated genes are potential prognostic factors, we performed a LASSO-Cox regression analysis on the DEGs (Figure 1b, c). We identified five genes, namely RRM2, FTCD, CYP2C9, ATP6V1C1, and POLD1, as potentially related to survival and may have obvious effects on the prognosis of HCC. Using univariate and multivariate Cox regression analysis, we analyzed the prognostic value of the methylation status and expression levels of the five aforementioned genes in HCC (Figure 1d, e), and obtained a gene signature, comprised of four genes, namely RRM2, FTCD, CYP2C9, and ATP6V1C1, that was strongly associated with HCC prognosis (*P* < 0.05). We found that the gene expression of RRM2 and ATP6V1C1 were upregulated in HCC patients, while that of FTCD and CYP2C9 were downregulated.

### Construction of the prognostic model

**Figure 2.**
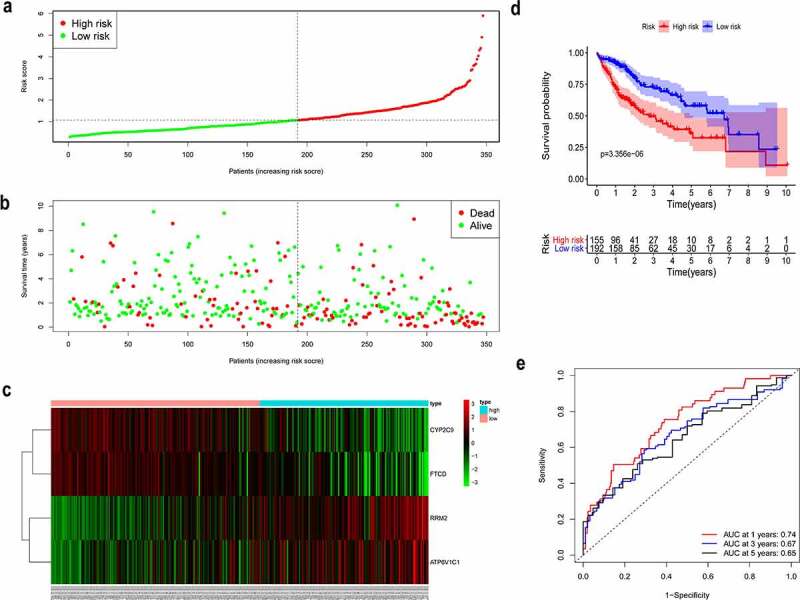
Construction of the prognostic model

To comprehensively represent the potential prognostic value of the four genes in HCC, a prognostic model was constructed based on the expression levels and the corresponding coefficients of the four genes and was used to calculate a risk score for patients with HCC from TCGA. Using the median value of the risk score, patients with HCC were divided into high-risk and low-risk groups (Figure 2a). As presented in Figure 2b, high-risk patients showed a shorter survival time than that of low-risk patients. The corresponding distributions of these prognostic factors were visualized by a heatmap (Figure 2c), which revealed that these prognostic factors were correlated with OS of patients with HCC.

(a-b) High-risk patients with HCC were correlated with a higher death rate and shorter survival time. (c) Heatmap depicting the expression levels of the four genes in HCC tissue. (d) The KM curve depicting the OS of the patient cohort from TCGA. (e) A time-dependent receiver operating characteristic curve depicting the 1-, 3-, and 5-year OS events of the patient cohort from TCGA.

Afterward, in order to assess the integrated effects of the low- and high-risk groups on HCC prognosis, a KM curve for OS was generated for the patient cohort from TCGA. Patients in high-risk group exhibited a shorter survival probability than those in low-risk group (Figure 2d, *P* = 3.356e-06). Furthermore, time-dependent ROC curve showed that the area under the curve (AUC) is around 0.74 at 1 year, 0.67 at 3 years, and 0.65 at 5 years (Figure 2e). Thus, the results showed that our prognostic model was efficacious in predicting the survival of HCC patients.

### Multivariate Cox regression analysis of the identified gene signature

To determine whether the identified gene signature is as an independent factor for the prognosis of HCC, we used the multivariate Cox regression analysis to analyze the patient cohort from TCGA. The multivariate Cox regression analysis involved the risk score, calculated using the gene signature, and clinical information of HCC patients, such as gender, age, grade, tumor, node and metastasis stage, and pathological stage. Importantly, the results indicated that the risk score was an independent variable correlated with the prognosis in HCC patients (Table 1, *P* = 2.8e-06).

**Table 1.** Multivariate Cox regression analysis of the gene signature in HCC patients

Variables		Univariate analysis			Multivariate analysis	
	HR (95%Cl)	Coefficient	*P*-value	HR (95%Cl)	Coefficient	*P*-value
Age Gender Gender male Histologic grade Pathologic T Pathologic N Pathologic stage Risk score	1(0.99–1) 0.73(0.47–1.1) NA 1.1(0.79–1.4) 1.7(1.4–2.1) 2(0.48–8) 1.7(1.4–2.2) 1.8(1.5–2.2)	0.0081 –0.31 NA 0.056 0.52 0.67 0.55 0.59	0.35 0.16 NA 0.71 **2.3e-06** **2.3e-09** **3.7e-06** **2.3e-09**	1(1–1) NA 0.88(0.55–1.4) 0.99(0.73–1.4) 1.6(0.65–3.9) 1.8(0.27–12) 0.97(0.37–2.6) 1.7(1.4–2.1)	0.014 NA -0.13 –0.0053 0.46 0.57 –0.026 0.52	0.13 NA 0.59 0.97 0.31 0.55 0.96 **2.8e-06**

The risk score calculated using the gene signature was found to be an independent variable correlated with the prognosis of HCC (*P* = 2.8e-06).

HR, hazard ratio; CI, confidence interval. Values in bold indicate significant P-value < 0.05.

### Validation of the gene signature in a patient cohort from the ICGC

**Figure 3.**
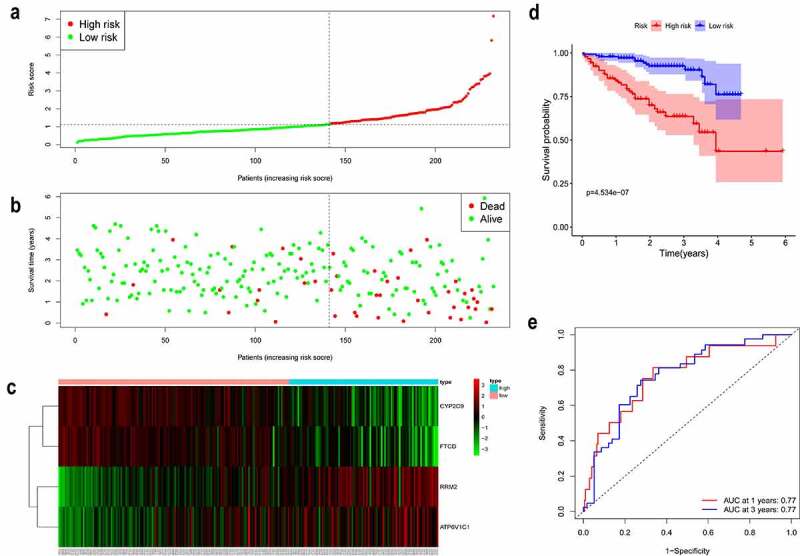
Validation of the gene signature in a patient cohort from the ICGC

We further demonstrated the reliability of the prognostic model in another independent patient cohort from the ICGC. Patients with HCC from the ICGC were classified into high- and low-risk groups by the median value of the risk score calculated using the gene expression levels and corresponding coefficients (Figure 3a). Consistent with the results in the TCGA cohort, high-risk patients showed a shorter survival time and poorer OS than that of low-risk patients (Figure 3b). The distribution of these prognostic factors in the ICGC cohort is presented in Figure 3c, further revealing that these iron metabolism-related and methylated genes are strongly correlated with the prognosis of HCC. The KM curve showed that patients in the high-risk group had shorter survival probability than those in the low-risk group (Figure 3d, *P* = 4.534e-06). The AUC was 0.77 at 1 year and 3 years (Figure 3e). In summary, these results validate our prognostic model.

(a-b) High-risk patients with HCC were correlated with a higher death rate and shorter survival time. (c) Heatmap depicting the expression levels of the four genes in HCC tissue. (d) The KM curve depicting the OS of the patient cohort from the ICGC. (e) A time-dependent receiver operating characteristic curve depicting the 1- and 3-year OS events of the patient cohort from the ICGC.

### Functional analysis of the iron metabolism-related and methylated genes

**Figure 4.**
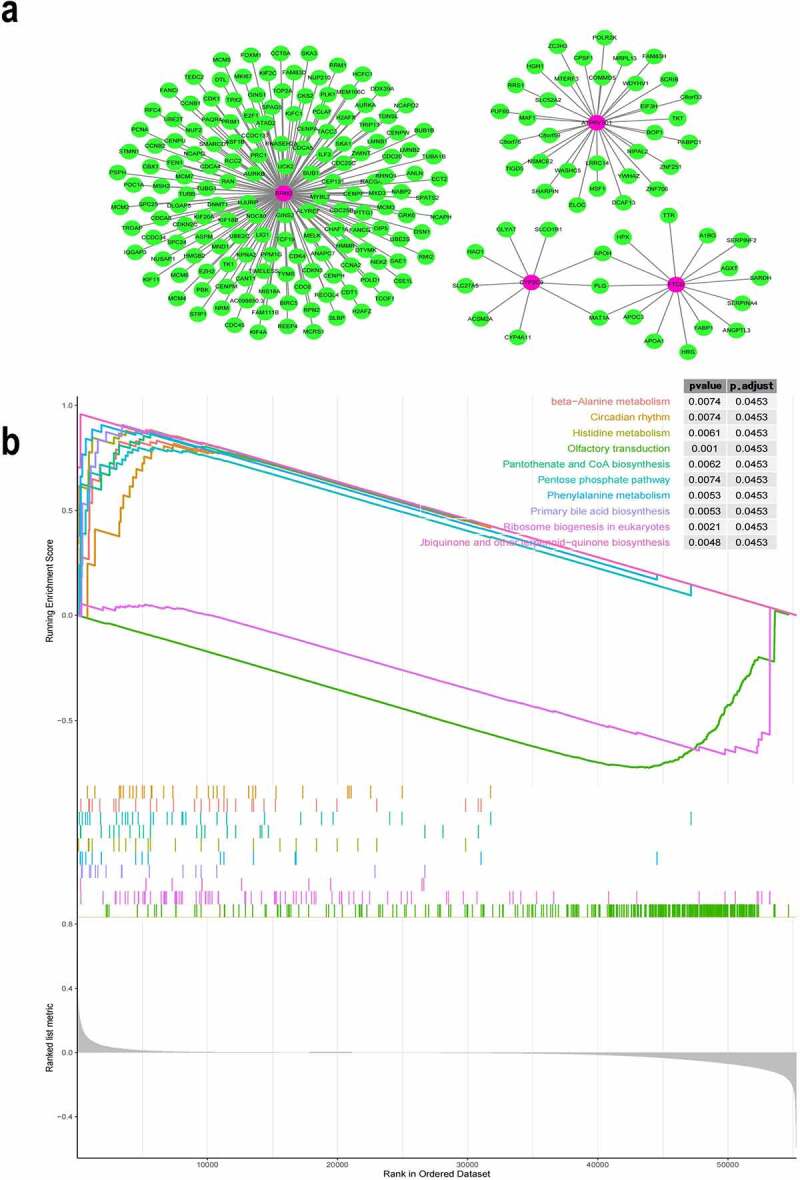
Co-expression network of the four iron metabolism-related and methylated genes

We explored the potential target genes that were regulated by the four genes of the prognostic model. We constructed a co-expression network involving the four genes using the WGCNA package in *R*. The co-expression network revealed the corresponding expression patterns of additional 213 target genes that were found to be co-expressed with the 4 genes (Figure 4a). Functional enrichment analysis, performed using GSEA showed that these DEGs identified in the high-risk group from TCGA were enriched in several important pathways, such as β-alanine metabolism, histidine metabolism, primary bile acid biosynthesis, phenylalanine metabolism, pentose phosphate pathway, pantothenate and coenzyme A biosynthesis, and ribosome biogenesis in eukaryotes (Figure 4b). Generally, these iron metabolism-related and methylated genes were associated with metabolism and biosynthesis in the pathogenesis of HCC.

(a)The co-expression network of the four genes that are a part of the identified gene signature. The rose red nodes denote the key genes, and the green nodes denote genes, which co-expressed with key genes. (b) Functional enrichment analysis with gene set enrichment analysis tool.

### Immunohistochemical results

**Figure 5.**
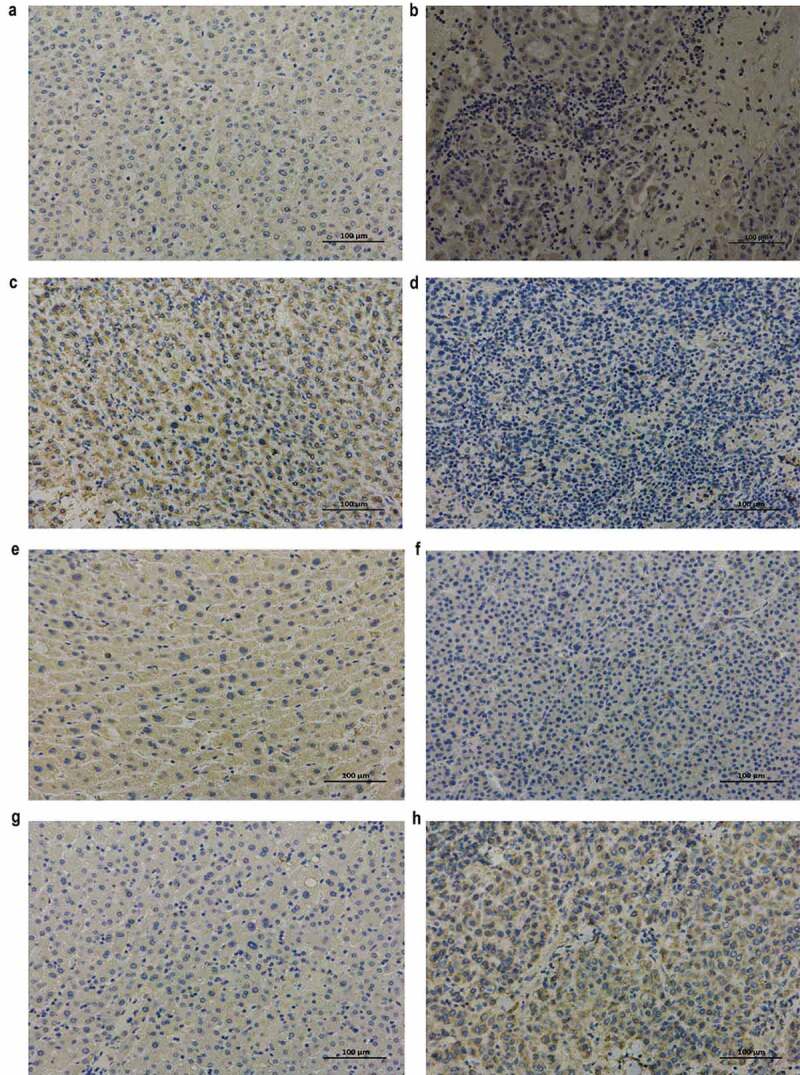
Immunohistochemical analysis of RRM2, FTCD, CYP2C9, and ATP6V1C1 in adjacent non-cancerous and cancerous tissues from HCC patients (original magnification, ×200)

The protein levels of RRM2 and ATP6V1C1 were increased and that of FTCD and CYP2C9 were decreased in cancerous tissues compared to that in adjacent non-cancerous tissue in HCC patients (Figure 5a-h), and this is consistent with our bioinformatic analysis. The results further validated the prognostic value of the four genes.

(a) RRM2 expression in adjacent non-cancerous tissue. (b) RRM2 expression in cancerous tissue. (c) FTCD expression in adjacent non-cancerous tissue. (d) FTCD expression in cancerous tissue. (e) CYP2C9 expression in adjacent non-cancerous tissue. (f) CYP2C9 expression in cancerous tissue. (g) ATP6V1C1 expression in adjacent non-cancerous tissue. (h) ATP6V1C1 expression in cancerous tissue.

## Discussion

HCC is a highly heterogeneous disease with a complex etiology and poor prognosis. In order to predict the prognosis of HCC, we identified a gene signature comprised of four genes related to iron metabolism and DNA methylation, namely RRM2, FTCD, CYP2C9, and ATP6V1C1, that was found to affect the prognosis of HCC. We constructed a novel prognostic model based on the expression of the aforementioned four genes and further validated it in an independent patient cohort.

Iron is an essential element involved in biological processes, such as DNA synthesis and repair, oxygen transport, cellular respiration, the activity of numerous enzymes, heme synthesis, xenobiotic metabolism, and immune function [11–13]. Compared with normal cells, tumor cells show an increased dependence on iron and are more susceptible to iron deficiency [14]. Therefore, iron metabolism disorder is widespread in patients with different kinds of cancers [15]. Previous research has demonstrated that the core of the iron metabolism disorder is oxidative stress, and the overload of intracellular iron catalyzes the excessive production of reactive oxygen species, leading to carcinogenesis or ferroptosis [16]. Of the genes found to be associated with the prognosis of HCC in the present study, RRM2, CYP2C9, and ATP6V1C1 are involved in the regulation of iron homeostasis, and FTCD affects iron metabolism by regulating the process of heme metabolism. Additionally, using bioinformatic analysis, we also found that the gene expression levels of RRM2 and ATP6V1C1 were upregulated in HCC patients, while that of FTCD and CYP2C9 were downregulated, and these results were consisted with our immunohistochemical results. However, due to the limited experimental sample size of our study, further studies are needed to support the findings.

Human ribonucleotide reductase (RR) is a heterotetrameric complex formed by two large subunits of RRM1 and two small subunits of RRM2. RR is the rate-limiting enzyme in DNA synthesis and repair and plays a critical role in cell proliferation and differentiation, making it an important anticancer target [17,18]. It has been observed that RRM2 overexpression in gastric, bladder, and colorectal cancers [19–21] dramatically enhances cellular invasiveness, angiogenesis, and metastasis [22]. Zhou et al. revealed that the upregulation of RRM2 is closely associated with the poor prognosis of HCC patients, and RRM2 is enriched in the p53 signaling pathway [23]. Moreover, Gao et al. showed that the expression of RRM2 was higher in HCC tissue than normal tissue, and an anti-RRM2 siRNA duplex exhibits anti-proliferative activity in HCC [24]. RRM2 has been reported to be an independent predictor of early recurrence of HCC, suggesting that RRM2 potentially promotes tumor cell metastasis [25].

To meet the high iron requirement for malignant cancers, the balance between iron import and export is inevitably changed. Studies have reported that iron overload is a common phenomenon in tumors, and thus, iron chelators are potential therapeutic agents against cancer. Triapine, a new-generation lipophilic iron chelator, has been identified to be one of the most potent inhibitors of RRM2 and may be a promising therapeutic strategy for cancers as well [26]. Sorafenib induces ferroptosis of cancer cells in HCC and is the first systemic molecular-targeted therapy drug approved by the FDA for advanced HCC [27]. Interestingly, RRM2 has been demonstrated as a novel target of sorafenib [28], and the inhibition of RRM2 activity significantly inhibited HCC progression [29]. These results demonstrate that therapeutic agents against HCC that affect iron levels also effect RRM2, indicating the role of RRM2 in iron metabolism in HCC.

FTCD is an intermediate metabolism enzyme composed of formiminotransferase and cyclodeaminase domains and is involved in the coupling of folate metabolism with histidine catabolism [30]. FTCD is abundantly expressed in human fetal and adult liver tissue and is associated with autoimmune hepatitis and glutamate formiminotransferase deficiency [31]. Consistent with our results, FTCD has been reported to be significantly downregulated in HCC tissues compared with nonneoplastic tissues [32]. In addition, Seimiya et al. showed that FTCD has a strong diagnostic potential for distinguishing early HCC [33]. Notably, the abnormal expression of FTCD is due to the high level of methylation in the promoter region of FTCD in HCC patients [34]. Thus, consistent with previous studies, we have demonstrated that the downregulation of FTCD is a prognostic factor of HCC, and FTCD can serve as a potential target for treatment of HCC.

CYP2C9, a member of the CYP450 family, is involved in a variety of biological processes, including drug metabolism and the synthesis of cholesterol, steroids, and lipids [35,36]. As one of the most abundant and important drug-metabolizing enzymes, the expression of CYP2C9 is associated with the risk of several diseases, such as lung cancer [37], colorectal cancer [38], and colorectal adenoma [39]. Suppression of CYP2C9 is closely correlated with the progression of HCC [40,41], and downregulation of CYP2C9 expression by hsa-miR-128-3p results in tumor cell invasion in HCC [42]. Importantly, activation of the CYP2C9 and STAT3 signaling pathway resulted in the sensitization of liver cancer stem cells to anticancer drugs, especially in advanced stages [43]. Thus, consistent with our findings, these reports indicate the impact of CYPC29 in HCC prognosis.

ATP6V1C1 encodes the V1 domain of the ATPase C subunit and is involved in pH regulation of intracellular processes. Over-expression of ATP6V1C1 has been observed in oral squamous cell carcinoma [44] and breast cancer [45], and it plays a major role in carcinogenesis. Furthermore, McConnell et al. reported that ATP6V1C1 enhanced tumor cell growth and metastasis by activating the mTORC1 pathway in breast cancer [46]. Studies have demonstrated the overexpression of ATP6L, which encodes the V0 domain of the V-ATPase C subunit, in HCC tissues and inhibitors of V-ATPase have been reported to significantly inhibit the growth of HCC tumor cells [47]. However, there are not many studies investigating the role of V-ATPase in the pathogenesis of HCC. To our knowledge, the present study is the first to report the overexpression of ATP6V1C1 in HCC, indicating that ATP6V1C1 may play an important role in the progression of HCC.

There are some bioinformatics studies that have investigated the prognosis of HCC prior to our research. Nevertheless, this study still has some obvious advantages. First, we have built a novel prognostic model for HCC integrating four iron metabolism-related and methylated gene, namely such as RRM2, FTCD, CYP2C9 and ATPV1C1, and our model showed potential clinical significance. Second, to our knowledge, we are the first report to demonstrate the prognostic value of ATP6V1C1 in HCC. However, there are also certain limitations in the present study. Our bioinformatic data are obtained from public databases, and the size and reliability of the data depend on the cohorts from TCGA and the ICGC. In addition, the AUC at 1, 3, 5 years for our prognostic model is about 0.73, 0.67, and 0.65, suggesting that we are still at a certain distance from the ideal model performance. Considering the complex biological process of HCC pathogenesis, we only focused on the prognostic role of iron metabolism-related and methylated genes; it is possible that prominent prognostic genes for HCC might have not been included in the prognostic model, thus reducing the performance of our model. Subsequent analyses involving larger sample sizes will aid in further uncovering underlying molecular mechanisms the prognosis of HCC.

## Conclusion

In conclusion, the present study identified genes related to iron metabolism and DNA methylation that are key to HCC prognosis. We also constructed a prognostic model that helped calculate risk scores based on the expression level of four genes. We found that the calculated risk score was an independent predictor of HCC prognosis. Despite its limitations, our results provide new insights into the prognosis of HCC.

## Supplementary Material

Supplemental Material

## Data Availability

The data that support the findings of the study are available in The Cancer Genome Atlas (https://cancergenome.nih.gov/), the Molecular Signatures Database (https://www.gsea-msigdb.org/gsea/msigdb), and the International Cancer Genome Consortium (https://dcc.icgc.org/).

## Highlights

1. Four genes involved in iron metabolism and DNA methylation are correlated with HCC prognosis.
2. The calculated risk score is an independent prognostic factor.
3. The four identified genes are potential treatment targets of HCC.
