# Supplementary material for: A novel four-gene of iron metabolism-related and methylated for prognosis prediction of hepatocellular carcinoma
Source: Bioengineered. 2020 Dec 31;12(1):240–51. doi: 10.1080/21655979.2020.1866303 (PMC8806199; doi:10.1080/21655979.2020.1866303)
Supplement: Supplemental Material [file KBIE_A_1866303_SM2202.zip › supplement/Supplementary Materials.docx]

**Supplementary Materials**

﻿Supplementary Table 1: Clinical information of hepatocellular carcinoma patients from The Cancer Genome Atlas.

Supplementary Table 2: Genes involve in iron metabolism obtained from the Molecular Signatures Database.

Supplementary Table 3: Differentially expressed genes between tumor tissue and normal tissue samples based on HCC transcriptomic data from The Cancer Genome Atlas.

Supplementary Table 4: Methylation status of differentially expressed iron metabolism-related genes.
