# Supplementary material for: A novel four-gene of iron metabolism-related and methylated for prognosis prediction of hepatocellular carcinoma
Source: Bioengineered. 2020 Dec 31;12(1):240–51. doi: 10.1080/21655979.2020.1866303 (PMC8806199; doi:10.1080/21655979.2020.1866303)
Supplement: Supplemental Material [file KBIE_A_1866303_SM2202.zip › supplement/Supplementary Table2.docx]

**Table S2**. Genes involve in iron metabolism obtained from the Molecular Signatures Database.

| id | SYMBLE |
| --- | --- |
| 1 | HFE |
| 2 | IFNG |
| 3 | ISCU |
| 4 | LTF |
| 5 | MIR210 |
| 6 | SLC11A2 |
| 7 | ACO1 |
| 8 | ACO2 |
| 9 | BRIP1 |
| 10 | CDK5RAP1 |
| 11 | CDKAL1 |
| 12 | CIAO3 |
| 13 | CIAPIN1 |
| 14 | DDX11 |
| 15 | DNA2 |
| 16 | DPYD |
| 17 | ELP3 |
| 18 | ERCC2 |
| 19 | ETFDH |
| 20 | EXO5 |
| 21 | IREB2 |
| 22 | ISCA2 |
| 23 | LIAS |
| 24 | MOCS1 |
| 25 | MUTYH |
| 26 | NDUFS1 |
| 27 | NDUFS2 |
| 28 | NDUFS7 |
| 29 | NDUFS8 |
| 30 | NDUFV1 |
| 31 | NFU1 |
| 32 | NTHL1 |
| 33 | NUBP1 |
| 34 | NUBP2 |
| 35 | NUBPL |
| 36 | POLA1 |
| 37 | POLD1 |
| 38 | POLE |
| 39 | PPAT |
| 40 | PRIM2 |
| 41 | REV3L |
| 42 | RSAD1 |
| 43 | RSAD2 |
| 44 | RTEL1 |
| 45 | SDHB |
| 46 | TYW1 |
| 47 | TYW1B |
| 48 | AIFM3 |
| 49 | AOX1 |
| 50 | CISD1 |
| 51 | CISD2 |
| 52 | CISD3 |
| 53 | FDX1 |
| 54 | FDX2 |
| 55 | FECH |
| 56 | FXN |
| 57 | GLRX2 |
| 58 | GLRX5 |
| 59 | ISCA1 |
| 60 | NDUFV2 |
| 61 | RFESD |
| 62 | UQCRFS1 |
| 63 | UQCRFS1P1 |
| 64 | XDH |
| 65 | ABCB6 |
| 66 | ATP7A |
| 67 | HAMP |
| 68 | MCOLN1 |
| 69 | MMGT1 |
| 70 | SCARA5 |
| 71 | SLC11A1 |
| 72 | SLC25A28 |
| 73 | SLC25A37 |
| 74 | SLC39A14 |
| 75 | SLC40A1 |
| 76 | STEAP2 |
| 77 | STEAP3 |
| 78 | STEAP4 |
| 79 | TF |
| 80 | TTYH1 |
| 81 | ABCB7 |
| 82 | ABCG2 |
| 83 | ALAS2 |
| 84 | ATP13A2 |
| 85 | ATP6AP1 |
| 86 | ATP6V0A2 |
| 87 | ATP6V0D1 |
| 88 | ATP6V1A |
| 89 | ATP6V1G1 |
| 90 | BMP6 |
| 91 | CAND1 |
| 92 | CCDC115 |
| 93 | CP |
| 94 | CUL1 |
| 95 | CYBRD1 |
| 96 | ERFE |
| 97 | FBXL5 |
| 98 | FLVCR1 |
| 99 | FTH1 |
| 100 | FTH1P19 |
| 101 | FTHL17 |
| 102 | FTL |
| 103 | FTMT |
| 104 | GDF2 |
| 105 | GLRX3 |
| 106 | HEPH |
| 107 | HIF1A |
| 108 | HJV |
| 109 | HMOX1 |
| 110 | HMOX2 |
| 111 | HPX |
| 112 | LCN2 |
| 113 | MYC |
| 114 | NCOA4 |
| 115 | NDFIP1 |
| 116 | NEDD8 |
| 117 | SKP1 |
| 118 | SLC22A17 |
| 119 | SLC46A1 |
| 120 | SMAD4 |
| 121 | SOD1 |
| 122 | SRI |
| 123 | TFR2 |
| 124 | TFRC |
| 125 | TMEM199 |
| 126 | TMPRSS6 |
| 127 | TTC7A |
| 128 | B2M |
| 129 | BDH2 |
| 130 | BTBD9 |
| 131 | EIF2AK1 |
| 132 | EPAS1 |
| 133 | EPB42 |
| 134 | HEPHL1 |
| 135 | HYAL2 |
| 136 | MELTF |
| 137 | NEO1 |
| 138 | PICALM |
| 139 | RHAG |
| 140 | STEAP1 |
| 141 | ASIC3 |
| 142 | FLVCR2 |
| 143 | HRG |
| 144 | SLC48A1 |
| 145 | MT2A |
| 146 | ACKR1 |
| 147 | ACP5 |
| 148 | ACSL6 |
| 149 | ADD1 |
| 150 | ADD2 |
| 151 | ADIPOR1 |
| 152 | AGPAT4 |
| 153 | AHSP |
| 154 | ALAD |
| 155 | ALDH1L1 |
| 156 | ALDH6A1 |
| 157 | ANK1 |
| 158 | AQP3 |
| 159 | ARHGEF12 |
| 160 | ARL2BP |
| 161 | ASNS |
| 162 | ATG4A |
| 163 | ATP6V0A1 |
| 164 | BACH1 |
| 165 | BCAM |
| 166 | BLVRA |
| 167 | BLVRB |
| 168 | BMP2K |
| 169 | BNIP3L |
| 170 | BPGM |
| 171 | BSG |
| 172 | BTG2 |
| 173 | BTRC |
| 174 | C3 |
| 175 | CA1 |
| 176 | CA2 |
| 177 | CAST |
| 178 | CAT |
| 179 | CCDC28A |
| 180 | CCND3 |
| 181 | CDC27 |
| 182 | CDR2 |
| 183 | CIR1 |
| 184 | CLCN3 |
| 185 | CLIC2 |
| 186 | CPOX |
| 187 | CROCCP2 |
| 188 | CTNS |
| 189 | CTSB |
| 190 | CTSE |
| 191 | DAAM1 |
| 192 | DCAF10 |
| 193 | DCAF11 |
| 194 | DCUN1D1 |
| 195 | DMTN |
| 196 | E2F2 |
| 197 | ELL2 |
| 198 | ENDOD1 |
| 199 | EPB41 |
| 200 | EPOR |
| 201 | ERMAP |
| 202 | EZH1 |
| 203 | FBXO34 |
| 204 | FBXO7 |
| 205 | FBXO9 |
| 206 | FN3K |
| 207 | FOXJ2 |
| 208 | FOXO3 |
| 209 | FTCD |
| 210 | GAPVD1 |
| 211 | GATA1 |
| 212 | GCLC |
| 213 | GCLM |
| 214 | GDE1 |
| 215 | GMPS |
| 216 | GYPA |
| 217 | GYPB |
| 218 | GYPC |
| 219 | GYPE |
| 220 | H1-0 |
| 221 | H4C3 |
| 222 | HAGH |
| 223 | HBB |
| 224 | HBBP1 |
| 225 | HBD |
| 226 | HBQ1 |
| 227 | HBZ |
| 228 | HDGF |
| 229 | HEBP1 |
| 230 | HMBS |
| 231 | HTATIP2 |
| 232 | HTRA2 |
| 233 | ICAM4 |
| 234 | IGSF3 |
| 235 | KAT2B |
| 236 | KDM7A |
| 237 | KEL |
| 238 | KHNYN |
| 239 | KLF1 |
| 240 | KLF3 |
| 241 | LAMP2 |
| 242 | LMO2 |
| 243 | LPIN2 |
| 244 | LRP10 |
| 245 | MAP2K3 |
| 246 | MARCHF2 |
| 247 | MARCHF8 |
| 248 | MARK3 |
| 249 | MBOAT2 |
| 250 | MFHAS1 |
| 251 | MGST3 |
| 252 | MINPP1 |
| 253 | MKRN1 |
| 254 | MOCOS |
| 255 | MOSPD1 |
| 256 | MPP1 |
| 257 | MXI1 |
| 258 | MYL4 |
| 259 | NARF |
| 260 | NEK7 |
| 261 | NFE2 |
| 262 | NFE2L1 |
| 263 | NNT |
| 264 | NR3C1 |
| 265 | NUDT4 |
| 266 | OPTN |
| 267 | OSBP2 |
| 268 | P4HA2 |
| 269 | PC |
| 270 | PDZK1IP1 |
| 271 | PGLS |
| 272 | PIGQ |
| 273 | PPOX |
| 274 | PPP2R5B |
| 275 | PRDX2 |
| 276 | PSMD9 |
| 277 | RAD23A |
| 278 | RANBP10 |
| 279 | RAP1GAP |
| 280 | RBM38 |
| 281 | RBM5 |
| 282 | RCL1 |
| 283 | RHCE |
| 284 | RHD |
| 285 | RIOK3 |
| 286 | RNF123 |
| 287 | RNF19A |
| 288 | SDCBP |
| 289 | SEC14L1 |
| 290 | SELENBP1 |
| 291 | SIDT2 |
| 292 | SLC10A3 |
| 293 | SLC22A4 |
| 294 | SLC25A38 |
| 295 | SLC2A1 |
| 296 | SLC30A1 |
| 297 | SLC30A10 |
| 298 | SLC4A1 |
| 299 | SLC66A2 |
| 300 | SLC6A8 |
| 301 | SLC6A9 |
| 302 | SLC7A11 |
| 303 | SMOX |
| 304 | SNCA |
| 305 | SPTA1 |
| 306 | SPTB |
| 307 | SYNJ1 |
| 308 | TAL1 |
| 309 | TCEA1 |
| 310 | TENT5C |
| 311 | TFDP2 |
| 312 | TMCC2 |
| 313 | TMEM9B |
| 314 | TNRC6B |
| 315 | TNS1 |
| 316 | TOP1 |
| 317 | TRAK2 |
| 318 | TRIM10 |
| 319 | TRIM58 |
| 320 | TSPAN5 |
| 321 | TSPO2 |
| 322 | TYR |
| 323 | UBAC1 |
| 324 | UCP2 |
| 325 | UROD |
| 326 | UROS |
| 327 | USP15 |
| 328 | VEZF1 |
| 329 | XK |
| 330 | XPO7 |
| 331 | YPEL5 |
| 332 | ATP6V0A4 |
| 333 | ATP6V0B |
| 334 | ATP6V0C |
| 335 | ATP6V0D2 |
| 336 | ATP6V0E1 |
| 337 | ATP6V0E2 |
| 338 | ATP6V1B1 |
| 339 | ATP6V1B2 |
| 340 | ATP6V1C1 |
| 341 | ATP6V1C2 |
| 342 | ATP6V1D |
| 343 | ATP6V1E1 |
| 344 | ATP6V1E2 |
| 345 | ATP6V1F |
| 346 | ATP6V1G2 |
| 347 | ATP6V1G3 |
| 348 | ATP6V1H |
| 349 | RPS27A |
| 350 | TCIRG1 |
| 351 | UBA52 |
| 352 | UBB |
| 353 | UBC |
| 354 | ALAS1 |
| 355 | COX10 |
| 356 | COX15 |
| 357 | TSPO |
| 358 | AMBP |
| 359 | ATP5IF1 |
| 360 | IBA57 |
| 361 | SLC25A39 |
| 362 | SRRD |
| 363 | TMEM14C |
| 364 | UGT1A1 |
| 365 | UGT1A4 |
| 366 | ABAT |
| 367 | APBB1 |
| 368 | BCL2 |
| 369 | BECN1 |
| 370 | C1QA |
| 371 | CCNB1 |
| 372 | CCND1 |
| 373 | CYP1A1 |
| 374 | DRD2 |
| 375 | G6PD |
| 376 | MAP1LC3A |
| 377 | MDM2 |
| 378 | PDX1 |
| 379 | SLC6A3 |
| 380 | TFAP2A |
| 381 | TFF1 |
| 382 | ARHGAP1 |
| 383 | CLTC |
| 384 | DNM2 |
| 385 | LMTK2 |
| 386 | NECTIN1 |
| 387 | RAB11B |
| 388 | REP15 |
| 389 | SFXN1 |
| 390 | ABCE1 |
| 391 | ADI1 |
| 392 | AGMO |
| 393 | ALKBH1 |
| 394 | ALKBH2 |
| 395 | ALKBH3 |
| 396 | ALKBH8 |
| 397 | ALOX12 |
| 398 | ALOX12B |
| 399 | ALOX15 |
| 400 | ALOX15B |
| 401 | ALOX5 |
| 402 | ALOXE3 |
| 403 | BBOX1 |
| 404 | CALR |
| 405 | CDO1 |
| 406 | CH25H |
| 407 | CYGB |
| 408 | CYP11A1 |
| 409 | CYP11B1 |
| 410 | CYP11B2 |
| 411 | CYP17A1 |
| 412 | CYP19A1 |
| 413 | CYP1A2 |
| 414 | CYP1B1 |
| 415 | CYP20A1 |
| 416 | CYP21A2 |
| 417 | CYP24A1 |
| 418 | CYP26A1 |
| 419 | CYP26B1 |
| 420 | CYP26C1 |
| 421 | CYP27A1 |
| 422 | CYP27B1 |
| 423 | CYP27C1 |
| 424 | CYP2A13 |
| 425 | CYP2A6 |
| 426 | CYP2A7 |
| 427 | CYP2B6 |
| 428 | CYP2C18 |
| 429 | CYP2C19 |
| 430 | CYP2C8 |
| 431 | CYP2C9 |
| 432 | CYP2D6 |
| 433 | CYP2D7 |
| 434 | CYP2E1 |
| 435 | CYP2F1 |
| 436 | CYP2G1P |
| 437 | CYP2J2 |
| 438 | CYP2R1 |
| 439 | CYP2S1 |
| 440 | CYP2U1 |
| 441 | CYP2W1 |
| 442 | CYP39A1 |
| 443 | CYP3A4 |
| 444 | CYP3A43 |
| 445 | CYP3A5 |
| 446 | CYP3A7 |
| 447 | CYP46A1 |
| 448 | CYP4A11 |
| 449 | CYP4A22 |
| 450 | CYP4B1 |
| 451 | CYP4F11 |
| 452 | CYP4F12 |
| 453 | CYP4F2 |
| 454 | CYP4F22 |
| 455 | CYP4F3 |
| 456 | CYP4F8 |
| 457 | CYP4V2 |
| 458 | CYP4X1 |
| 459 | CYP4Z1 |
| 460 | CYP4Z2P |
| 461 | CYP51A1 |
| 462 | CYP7A1 |
| 463 | CYP7B1 |
| 464 | CYP8B1 |
| 465 | DNAJC24 |
| 466 | DOHH |
| 467 | EGLN1 |
| 468 | EGLN2 |
| 469 | EGLN3 |
| 470 | ETHE1 |
| 471 | FA2H |
| 472 | FAXDC2 |
| 473 | FTO |
| 474 | HAAO |
| 475 | HBA1 |
| 476 | HBA2 |
| 477 | HIF1AN |
| 478 | JMJD6 |
| 479 | KDM3A |
| 480 | MIOX |
| 481 | MSMO1 |
| 482 | OGFOD1 |
| 483 | OGFOD2 |
| 484 | OGFOD3 |
| 485 | P3H1 |
| 486 | P3H2 |
| 487 | P3H3 |
| 488 | P4HA1 |
| 489 | P4HA3 |
| 490 | P4HTM |
| 491 | PAH |
| 492 | PHF2 |
| 493 | PHF8 |
| 494 | PHYH |
| 495 | PLOD1 |
| 496 | PLOD2 |
| 497 | PLOD3 |
| 498 | PPEF1 |
| 499 | PPEF2 |
| 500 | PTGIS |
| 501 | RIOX1 |
| 502 | RRM2 |
| 503 | SC5D |
| 504 | SCD |
| 505 | SCD5 |
| 506 | TBXAS1 |
| 507 | TET1 |
| 508 | TET2 |
| 509 | TET3 |
| 510 | TH |
| 511 | TMLHE |
| 512 | TPH1 |
| 513 | TPH2 |
| 514 | TYW5 |
