# Supplementary material for: A novel four-gene of iron metabolism-related and methylated for prognosis prediction of hepatocellular carcinoma
Source: Bioengineered. 2020 Dec 31;12(1):240–51. doi: 10.1080/21655979.2020.1866303 (PMC8806199; doi:10.1080/21655979.2020.1866303)
Supplement: Supplemental Material [file KBIE_A_1866303_SM2202.zip › supplement/Supplementary Table3.docx]

**Table S3.** Differentially expressed genes between tumor tissue and normal tissue samples based on HCC transcriptomic data from The Cancer Genome Atlas.

|  | logFC | AveExpr | t | P.Value | adj.P.Val | B |
| --- | --- | --- | --- | --- | --- | --- |
| GDF2 | -2.59337 | 0.524507 | -28.1761 | 7.03E-99 | 3.58E-96 | 214.8431 |
| CDK5RAP1 | 0.830781 | 2.456708 | 16.20831 | 3.01E-46 | 7.67E-44 | 94.38568 |
| PPOX | 1.156811 | 2.146344 | 15.78121 | 2.14E-44 | 3.63E-42 | 90.14814 |
| HAMP | -4.75235 | 2.429867 | -15.7261 | 3.70E-44 | 4.71E-42 | 89.60293 |
| ATP6AP1 | 1.261921 | 4.66046 | 15.26533 | 3.55E-42 | 3.09E-40 | 85.0686 |
| PLOD3 | 1.445804 | 4.298722 | 15.25426 | 3.96E-42 | 3.09E-40 | 84.96007 |
| SLC25A39 | 1.275872 | 5.410859 | 15.24698 | 4.25E-42 | 3.09E-40 | 84.88874 |
| CYP26A1 | -1.77617 | 0.662865 | -14.9924 | 5.20E-41 | 3.31E-39 | 82.40035 |
| CYP1A2 | -5.02944 | 2.560852 | -14.5313 | 4.70E-39 | 2.66E-37 | 77.92606 |
| CCNB1 | 2.261678 | 2.617807 | 13.30337 | 5.99E-34 | 3.05E-32 | 66.25184 |
| ATP6V1E1 | 0.962361 | 4.596641 | 13.16589 | 2.18E-33 | 1.01E-31 | 64.96958 |
| BSG | 1.437186 | 6.519392 | 12.76351 | 9.25E-32 | 3.78E-30 | 61.2489 |
| TMEM199 | 0.722298 | 2.004009 | 12.759 | 9.64E-32 | 3.78E-30 | 61.20752 |
| ATP6V1F | 1.229529 | 5.994289 | 12.63606 | 3.00E-31 | 1.09E-29 | 60.08088 |
| RRM2 | 1.981539 | 2.230709 | 12.62447 | 3.34E-31 | 1.13E-29 | 59.97495 |
| CYP2R1 | 0.758061 | 1.579619 | 12.51514 | 9.12E-31 | 2.90E-29 | 58.97763 |
| RIOX1 | 0.80985 | 2.152861 | 12.48141 | 1.24E-30 | 3.72E-29 | 58.67073 |
| SLC6A3 | 0.075401 | 0.070991 | 1.447071 | 0.148622 | 0.183614 | -6.48126 |
| EZH1 | 0.817073 | 1.839858 | 12.40261 | 2.56E-30 | 6.85E-29 | 57.95521 |
| NEDD8 | 0.853838 | 3.845018 | 12.25832 | 9.52E-30 | 2.42E-28 | 56.65061 |
| BPGM | 0.93108 | 2.528825 | 12.11816 | 3.39E-29 | 8.22E-28 | 55.39016 |
| ATP6V1C1 | 1.284289 | 3.462969 | 12.05173 | 6.18E-29 | 1.43E-27 | 54.79525 |
| CYP2C19 | -1.17722 | 0.407012 | -12.0224 | 8.05E-29 | 1.78E-27 | 54.53338 |
| CYP39A1 | -2.35505 | 2.024083 | -11.9269 | 1.90E-28 | 3.87E-27 | 53.68176 |
| SKP1 | 0.743648 | 3.737053 | 11.92676 | 1.90E-28 | 3.87E-27 | 53.68026 |
| NARF | 1.067731 | 2.431327 | 11.8819 | 2.84E-28 | 5.57E-27 | 53.28143 |
| POLD1 | 1.221399 | 2.183163 | 11.86757 | 3.23E-28 | 6.10E-27 | 53.15424 |
| GLRX3 | 0.867922 | 2.86328 | 11.82275 | 4.83E-28 | 8.78E-27 | 52.75671 |
| PRIM2 | 0.845453 | 1.325604 | 11.80033 | 5.90E-28 | 1.04E-26 | 52.5582 |
| TMEM14C | 0.941044 | 5.956498 | 11.51973 | 7.12E-27 | 1.21E-25 | 50.08933 |
| CALR | 0.996711 | 8.778476 | 11.40602 | 1.94E-26 | 3.18E-25 | 49.09762 |
| ADIPOR1 | 0.848638 | 5.330623 | 11.29727 | 5.01E-26 | 7.98E-25 | 48.15405 |
| HMBS | 0.810701 | 2.62966 | 11.22053 | 9.79E-26 | 1.51E-24 | 47.49108 |
| MT2A | -3.12914 | 7.397054 | -11.1494 | 1.82E-25 | 2.72E-24 | 46.87839 |
| ISCA2 | 0.673171 | 2.364447 | 11.10105 | 2.76E-25 | 4.01E-24 | 46.46371 |
| TFRC | 1.240105 | 3.160179 | 9.380974 | 4.12E-19 | 3.18E-18 | 32.3966 |
| P3H1 | 0.920717 | 2.617513 | 10.89791 | 1.59E-24 | 2.18E-23 | 44.73082 |
| CYP2B6 | -2.96275 | 4.001944 | -10.8663 | 2.08E-24 | 2.79E-23 | 44.463 |
| MKRN1 | 0.690761 | 3.469313 | 10.83677 | 2.68E-24 | 3.50E-23 | 44.21278 |
| P4HA2 | 1.213209 | 2.04785 | 10.81562 | 3.21E-24 | 4.08E-23 | 44.03396 |
| DOHH | 0.776258 | 2.545509 | 10.72306 | 7.05E-24 | 8.76E-23 | 43.25366 |
| HIF1AN | 0.677473 | 1.478937 | 10.53944 | 3.33E-23 | 4.03E-22 | 41.7171 |
| ETFDH | -1.19597 | 3.499865 | -10.4931 | 4.91E-23 | 5.81E-22 | 41.3321 |
| ATP6V1H | 0.885429 | 2.760045 | 10.47639 | 5.65E-23 | 6.54E-22 | 41.19298 |
| CIAO3 | 0.614636 | 1.996684 | 10.45133 | 6.97E-23 | 7.89E-22 | 40.98525 |
| GMPS | 0.864545 | 2.38924 | 10.40448 | 1.03E-22 | 1.14E-21 | 40.59755 |
| HTRA2 | 0.67429 | 2.675032 | 10.37854 | 1.28E-22 | 1.39E-21 | 40.38335 |
| CYP4B1 | 0.018401 | 0.049468 | 1.035591 | 0.300988 | 0.346613 | -6.98987 |
| MCOLN1 | 0.708086 | 2.957942 | 10.19249 | 5.99E-22 | 6.22E-21 | 38.85627 |
| ATP6V1D | 0.628599 | 3.232247 | 10.1422 | 9.07E-22 | 9.23E-21 | 38.44631 |
| CYP2C8 | -3.3163 | 5.796417 | -10.0463 | 1.99E-21 | 1.99E-20 | 37.6677 |
| RBM5 | 0.652549 | 2.237546 | 9.90469 | 6.31E-21 | 6.18E-20 | 36.5271 |
| ALKBH2 | 1.022539 | 3.209492 | 9.894226 | 6.87E-21 | 6.60E-20 | 36.44319 |
| PPP2R5B | 0.701541 | 2.163814 | 9.84202 | 1.05E-20 | 9.88E-20 | 36.02537 |
| CIAPIN1 | 0.756349 | 3.154087 | 9.796024 | 1.52E-20 | 1.41E-19 | 35.6584 |
| MUTYH | 0.693383 | 1.66787 | 9.725607 | 2.68E-20 | 2.43E-19 | 35.09868 |
| NDUFS8 | 0.9005 | 4.16675 | 9.595884 | 7.54E-20 | 6.74E-19 | 34.07426 |
| RSAD1 | 0.653955 | 3.584415 | 9.581884 | 8.43E-20 | 7.40E-19 | 33.96423 |
| HYAL2 | 0.741059 | 3.295159 | 9.576239 | 8.82E-20 | 7.61E-19 | 33.91989 |
| HBB | -1.95823 | 3.154217 | -9.55651 | 1.03E-19 | 8.75E-19 | 33.76503 |
| CYP4A22 | -2.23802 | 3.855459 | -9.50958 | 1.50E-19 | 1.25E-18 | 33.39756 |
| ATP6V0B | 0.893636 | 5.018507 | 9.50198 | 1.59E-19 | 1.30E-18 | 33.3382 |
| SCD | 0.349967 | 7.442019 | 1.324459 | 0.18607 | 0.223372 | -6.65031 |
| ATP6V0E1 | 0.808945 | 6.343588 | 9.446539 | 2.46E-19 | 1.96E-18 | 32.90582 |
| OPTN | 0.882482 | 4.39719 | 9.395321 | 3.68E-19 | 2.88E-18 | 32.50784 |
| ATP6V0A4 | 0.015236 | 0.018152 | 1.233987 | 0.217897 | 0.258531 | -6.76554 |
| HDGF | 0.726179 | 6.49707 | 9.376972 | 4.25E-19 | 3.23E-18 | 32.3656 |
| FBXO9 | 0.783616 | 2.587466 | 9.297893 | 7.89E-19 | 5.90E-18 | 31.75468 |
| CDKAL1 | 0.644984 | 1.830976 | 9.217478 | 1.47E-18 | 1.09E-17 | 31.13694 |
| G6PD | 1.832051 | 2.778823 | 9.215148 | 1.50E-18 | 1.09E-17 | 31.11909 |
| ATP13A2 | 0.927976 | 1.94328 | 9.190835 | 1.81E-18 | 1.30E-17 | 30.93305 |
| SLC46A1 | 0.777129 | 2.108032 | 9.170316 | 2.12E-18 | 1.50E-17 | 30.7763 |
| SLC22A17 | 0.203421 | 0.965311 | 1.203239 | 0.229561 | 0.269853 | -6.80286 |
| CYP3A4 | -4.57472 | 5.283584 | -9.01222 | 7.16E-18 | 4.93E-17 | 29.57643 |
| JMJD6 | 0.736078 | 2.119224 | 9.007989 | 7.40E-18 | 5.02E-17 | 29.54447 |
| PPAT | 0.492536 | 1.318545 | 6.965188 | 1.27E-11 | 4.49E-11 | 15.4167 |
| USP15 | 0.054471 | 1.273653 | 1.28514 | 0.199451 | 0.238311 | -6.70138 |
| UBAC1 | 0.588886 | 3.218381 | 8.927254 | 1.37E-17 | 8.93E-17 | 28.93734 |
| GLRX2 | 0.778925 | 3.090377 | 8.88666 | 1.86E-17 | 1.20E-16 | 28.63348 |
| SLC48A1 | 0.721005 | 2.505003 | 8.868847 | 2.13E-17 | 1.36E-16 | 28.50044 |
| ALDH6A1 | -1.55753 | 4.606879 | -8.86576 | 2.18E-17 | 1.37E-16 | 28.47739 |
| STEAP4 | -1.03605 | 1.001919 | -8.80561 | 3.43E-17 | 2.13E-16 | 28.02965 |
| NECTIN1 | 0.902808 | 1.483924 | 8.699324 | 7.62E-17 | 4.67E-16 | 27.24352 |
| MGST3 | 0.664007 | 3.283074 | 8.165439 | 3.77E-15 | 1.86E-14 | 23.39698 |
| SLC39A14 | -1.21688 | 5.282279 | -8.64099 | 1.18E-16 | 7.04E-16 | 26.81492 |
| CIR1 | 0.56536 | 3.0759 | 8.626683 | 1.31E-16 | 7.74E-16 | 26.71007 |
| HBA2 | -1.64018 | 2.458092 | -8.62421 | 1.33E-16 | 7.79E-16 | 26.692 |
| UBA52 | 0.832523 | 6.412625 | 8.554863 | 2.23E-16 | 1.29E-15 | 26.18573 |
| SRRD | 0.5134 | 1.772281 | 8.552771 | 2.26E-16 | 1.29E-15 | 26.1705 |
| RPS27A | 0.917164 | 6.634681 | 8.520685 | 2.86E-16 | 1.62E-15 | 25.93728 |
| EGLN2 | 0.753664 | 2.012911 | 8.516286 | 2.96E-16 | 1.65E-15 | 25.90536 |
| CYP4Z1 | -0.01945 | 0.115063 | -0.67657 | 0.499049 | 0.548631 | -7.29652 |
| CLTC | 0.723661 | 4.650244 | 8.381324 | 7.95E-16 | 4.35E-15 | 24.93154 |
| DDX11 | 0.730752 | 1.091301 | 8.348894 | 1.01E-15 | 5.45E-15 | 24.69918 |
| ATP6V0D1 | 0.644764 | 3.320097 | 8.341837 | 1.06E-15 | 5.67E-15 | 24.6487 |
| ARHGAP1 | 0.724933 | 3.486528 | 8.285756 | 1.59E-15 | 8.42E-15 | 24.24867 |
| B2M | 0.117127 | 8.900817 | 0.92185 | 0.357136 | 0.406671 | -7.10089 |
| YPEL5 | 0.578327 | 4.257422 | 8.274562 | 1.72E-15 | 8.95E-15 | 24.16905 |
| CTNS | 0.560307 | 2.475551 | 8.250659 | 2.05E-15 | 1.05E-14 | 23.9993 |
| BLVRA | 1.34112 | 3.151178 | 8.234858 | 2.29E-15 | 1.17E-14 | 23.88728 |
| TMEM9B | 0.558507 | 3.478914 | 8.19427 | 3.07E-15 | 1.53E-14 | 23.60025 |
| EXO5 | 0.525437 | 1.288405 | 8.194209 | 3.07E-15 | 1.53E-14 | 23.59982 |
| MBOAT2 | 0.082503 | 0.30744 | 1.224834 | 0.221324 | 0.261729 | -6.77675 |
| PGLS | 0.774627 | 3.975161 | 8.148956 | 4.24E-15 | 2.08E-14 | 23.28101 |
| OGFOD2 | 0.21009 | 0.442886 | 8.113105 | 5.48E-15 | 2.66E-14 | 23.02935 |
| POLA1 | 0.626373 | 1.159215 | 8.088194 | 6.54E-15 | 3.14E-14 | 22.85495 |
| NDUFS2 | 0.616412 | 5.612093 | 8.041029 | 9.14E-15 | 4.35E-14 | 22.52585 |
| ENDOD1 | -0.13486 | 1.556758 | -1.04615 | 0.296095 | 0.341751 | -6.97892 |
| MARK3 | 0.478694 | 2.151653 | 8.033662 | 9.63E-15 | 4.50E-14 | 22.47457 |
| SLC40A1 | -0.12168 | 5.401788 | -0.91358 | 0.361462 | 0.410679 | -7.10846 |
| ALOX5 | 0.194185 | 1.124877 | 1.317579 | 0.188362 | 0.225591 | -6.65936 |
| TCEA1 | 0.699288 | 3.592378 | 7.952175 | 1.71E-14 | 7.77E-14 | 21.90968 |
| RCL1 | -0.98015 | 2.842243 | -7.84225 | 3.68E-14 | 1.66E-13 | 21.15444 |
| CYP4V2 | -0.96596 | 3.105738 | -7.82779 | 4.07E-14 | 1.81E-13 | 21.05561 |
| FDX2 | 0.595456 | 1.333642 | 7.827409 | 4.08E-14 | 1.81E-13 | 21.05303 |
| KHNYN | 0.751805 | 2.02781 | 7.820827 | 4.27E-14 | 1.88E-13 | 21.00812 |
| FTH1 | 0.870382 | 7.857623 | 7.811757 | 4.55E-14 | 1.98E-13 | 20.94627 |
| OGFOD3 | 0.556798 | 2.512546 | 7.751794 | 6.89E-14 | 2.97E-13 | 20.53877 |
| NFU1 | 0.53611 | 3.179367 | 7.739939 | 7.47E-14 | 3.20E-13 | 20.45848 |
| BBOX1 | -1.70689 | 1.984455 | -7.72439 | 8.32E-14 | 3.53E-13 | 20.35328 |
| TTC7A | 0.716807 | 2.221514 | 7.680434 | 1.12E-13 | 4.73E-13 | 20.05687 |
| HTATIP2 | 0.97116 | 4.828945 | 7.646419 | 1.42E-13 | 5.92E-13 | 19.82835 |
| KDM3A | 0.517892 | 1.504406 | 7.042113 | 7.77E-12 | 2.79E-11 | 15.89877 |
| CYP2C9 | -2.39297 | 5.990065 | -7.55686 | 2.61E-13 | 1.07E-12 | 19.23037 |
| BTBD9 | 0.406046 | 1.049295 | 7.464868 | 4.84E-13 | 1.97E-12 | 18.62167 |
| ABAT | -1.41937 | 4.464895 | -7.45115 | 5.31E-13 | 2.15E-12 | 18.53141 |
| FXN | -0.56605 | 2.263305 | -7.41833 | 6.61E-13 | 2.65E-12 | 18.31592 |
| ADD1 | 0.579079 | 3.368676 | 7.352196 | 1.03E-12 | 4.08E-12 | 17.88396 |
| CDC27 | 0.54498 | 2.618144 | 7.339435 | 1.12E-12 | 4.41E-12 | 17.80095 |
| ATP6V1A | 0.557274 | 3.619878 | 7.312549 | 1.34E-12 | 5.23E-12 | 17.62643 |
| ASIC3 | 0.466614 | 0.541766 | 7.290824 | 1.54E-12 | 5.99E-12 | 17.48577 |
| IGSF3 | 1.035025 | 1.206846 | 7.235889 | 2.21E-12 | 8.51E-12 | 17.13154 |
| CAT | -1.02473 | 6.46509 | -7.23497 | 2.22E-12 | 8.51E-12 | 17.12561 |
| TYW1 | 0.421595 | 2.249237 | 7.184805 | 3.09E-12 | 1.17E-11 | 16.80401 |
| PLOD1 | 0.755694 | 5.238317 | 7.136877 | 4.22E-12 | 1.59E-11 | 16.49836 |
| BRIP1 | 0.454186 | 0.600698 | 7.134724 | 4.27E-12 | 1.60E-11 | 16.48467 |
| ATP6V1C2 | 0.321728 | 0.475061 | 7.133145 | 4.32E-12 | 1.60E-11 | 16.47463 |
| APBB1 | 0.642382 | 1.599567 | 7.116244 | 4.82E-12 | 1.78E-11 | 16.36728 |
| HPX | -1.89903 | 8.659741 | -7.07255 | 6.39E-12 | 2.34E-11 | 16.09067 |
| TET3 | 0.501863 | 0.993152 | 7.055038 | 7.15E-12 | 2.60E-11 | 15.98018 |
| AQP3 | -1.19061 | 4.03821 | -7.0437 | 7.69E-12 | 2.78E-11 | 15.90875 |
| ATP6V0C | 0.580136 | 4.002581 | 5.067521 | 6.05E-07 | 1.39E-06 | 4.930826 |
| MOSPD1 | 0.749436 | 1.974827 | 7.024904 | 8.68E-12 | 3.09E-11 | 15.79056 |
| RFESD | 0.149524 | 0.317985 | 8.015689 | 1.09E-14 | 5.06E-14 | 22.34961 |
| BDH2 | -0.78489 | 2.782286 | -6.92922 | 1.60E-11 | 5.61E-11 | 15.19274 |
| CYP2A6 | -3.36264 | 5.805575 | -6.91591 | 1.74E-11 | 6.04E-11 | 15.1101 |
| LRP10 | 0.766149 | 3.789771 | 6.91539 | 1.74E-11 | 6.04E-11 | 15.10686 |
| EGLN1 | 0.611683 | 3.621986 | 6.867795 | 2.35E-11 | 8.10E-11 | 14.81238 |
| CYP4F2 | -1.74063 | 4.325932 | -6.8528 | 2.59E-11 | 8.84E-11 | 14.71995 |
| CYP8B1 | -2.57912 | 4.7388 | -6.83195 | 2.95E-11 | 1.00E-10 | 14.59168 |
| PTGIS | -0.82298 | 0.853905 | -6.82543 | 3.07E-11 | 1.04E-10 | 14.55164 |
| IBA57 | 0.337983 | 1.072848 | 6.803847 | 3.52E-11 | 1.18E-10 | 14.41928 |
| CYP2E1 | -3.24768 | 5.97469 | -6.7963 | 3.69E-11 | 1.23E-10 | 14.37311 |
| SLC10A3 | 0.749342 | 2.514226 | 6.742477 | 5.16E-11 | 1.70E-10 | 14.0448 |
| ALOX15 | 0.071199 | 0.093657 | 2.340088 | 0.019746 | 0.027612 | -4.80618 |
| ATP6V1E2 | 0.355764 | 0.628851 | 6.627282 | 1.05E-10 | 3.43E-10 | 13.34927 |
| SLC11A1 | 0.09375 | 0.390337 | 1.401817 | 0.161708 | 0.197384 | -6.54538 |
| BCAM | 1.172783 | 4.616791 | 6.602312 | 1.22E-10 | 3.94E-10 | 13.19979 |
| LMTK2 | 0.571092 | 1.964446 | 6.589373 | 1.32E-10 | 4.24E-10 | 13.1225 |
| HAAO | -1.10359 | 5.26516 | -6.53753 | 1.82E-10 | 5.78E-10 | 12.8141 |
| LCN2 | 2.649774 | 4.133534 | 6.52663 | 1.94E-10 | 6.13E-10 | 12.7495 |
| CYP3A43 | -0.86045 | 0.985632 | -6.43159 | 3.44E-10 | 1.08E-09 | 12.19003 |
| ISCU | 0.40644 | 3.900717 | 6.429333 | 3.49E-10 | 1.09E-09 | 12.17686 |
| EIF2AK1 | 0.649731 | 4.88012 | 10.23819 | 4.11E-22 | 4.36E-21 | 39.22985 |
| SLC4A1 | -0.02812 | 0.046126 | -0.84949 | 0.396092 | 0.446041 | -7.16482 |
| CYP2A7 | -2.18749 | 2.340021 | -6.35129 | 5.55E-10 | 1.70E-09 | 11.72265 |
| CA2 | -1.23522 | 4.302723 | -6.34335 | 5.82E-10 | 1.77E-09 | 11.67668 |
| TSPO2 | 0.546018 | 0.636345 | 6.308439 | 7.15E-10 | 2.17E-09 | 11.47518 |
| LIAS | 0.411149 | 2.322723 | 6.296271 | 7.68E-10 | 2.31E-09 | 11.40517 |
| HIF1A | 0.242844 | 3.419641 | 1.601761 | 0.109959 | 0.139227 | -6.24686 |
| CISD1 | 0.506672 | 3.302945 | 6.267094 | 9.12E-10 | 2.71E-09 | 11.23774 |
| OSBP2 | 0.495719 | 0.617757 | 6.266118 | 9.17E-10 | 2.71E-09 | 11.23215 |
| ATP6V0A2 | 0.240179 | 0.922882 | 6.228449 | 1.14E-09 | 3.36E-09 | 11.01697 |
| CP | -1.43785 | 6.136387 | -6.1885 | 1.44E-09 | 4.22E-09 | 10.78992 |
| FTMT | 0.003138 | 0.002766 | 0.602918 | 0.546889 | 0.586034 | -7.34357 |
| CISD2 | 0.40675 | 3.99085 | 6.117901 | 2.17E-09 | 6.28E-09 | 10.39172 |
| TFDP2 | 0.413809 | 1.574743 | 6.710084 | 6.31E-11 | 2.07E-10 | 13.84824 |
| AOX1 | -1.77473 | 6.135232 | -6.06584 | 2.93E-09 | 8.37E-09 | 10.1005 |
| ABCB6 | 0.53931 | 1.549455 | 6.055559 | 3.11E-09 | 8.83E-09 | 10.04324 |
| CUL1 | 0.442393 | 4.189167 | 6.027643 | 3.64E-09 | 1.03E-08 | 9.888167 |
| FOXJ2 | 0.390703 | 1.51321 | 5.962976 | 5.25E-09 | 1.48E-08 | 9.531263 |
| ALAS1 | -0.96571 | 6.312346 | -5.95642 | 5.45E-09 | 1.52E-08 | 9.495286 |
| SLC6A9 | 0.624011 | 0.762137 | 5.927289 | 6.42E-09 | 1.79E-08 | 9.335692 |
| ERFE | 0.384735 | 0.399589 | 5.902057 | 7.40E-09 | 2.05E-08 | 9.198017 |
| SDCBP | 0.668444 | 4.697704 | 5.891575 | 7.85E-09 | 2.16E-08 | 9.140967 |
| XDH | -1.17703 | 2.915544 | -5.88957 | 7.93E-09 | 2.17E-08 | 9.130088 |
| UROS | 0.443848 | 2.670902 | 5.871369 | 8.78E-09 | 2.39E-08 | 9.031239 |
| MMGT1 | 0.499507 | 2.212887 | 5.865366 | 9.08E-09 | 2.46E-08 | 8.998703 |
| VEZF1 | 0.511193 | 2.403292 | 5.829413 | 1.11E-08 | 2.99E-08 | 8.80442 |
| AIFM3 | 0.299931 | 0.409834 | 5.758574 | 1.64E-08 | 4.39E-08 | 8.424601 |
| POLE | 0.406186 | 1.546103 | 5.757891 | 1.65E-08 | 4.39E-08 | 8.420958 |
| SCARA5 | -0.43917 | 0.30328 | -5.72055 | 2.02E-08 | 5.35E-08 | 8.222365 |
| UGT1A1 | -1.49415 | 3.228582 | -5.69808 | 2.28E-08 | 6.02E-08 | 8.103382 |
| RAB11B | 0.41222 | 5.26229 | 5.639811 | 3.13E-08 | 8.21E-08 | 7.796727 |
| NFE2L1 | 0.594122 | 5.542288 | 5.637652 | 3.17E-08 | 8.26E-08 | 7.785418 |
| HMOX1 | -1.02585 | 4.28951 | -5.63508 | 3.21E-08 | 8.34E-08 | 7.771944 |
| UGT1A4 | -1.79435 | 3.277881 | -5.6327 | 3.25E-08 | 8.40E-08 | 7.759499 |
| FTCD | -1.44578 | 5.933777 | -5.60055 | 3.87E-08 | 9.94E-08 | 7.591615 |
| UBC | 0.416373 | 6.763578 | 5.591591 | 4.06E-08 | 1.04E-07 | 7.545011 |
| ASNS | 0.848768 | 1.254582 | 5.514213 | 6.13E-08 | 1.56E-07 | 7.144972 |
| NDFIP1 | 0.3546 | 4.785293 | 5.468415 | 7.81E-08 | 1.98E-07 | 6.910472 |
| CYP2J2 | -0.92656 | 4.296272 | -5.44014 | 9.06E-08 | 2.28E-07 | 6.76655 |
| GAPVD1 | 0.283462 | 1.410604 | 5.418844 | 1.01E-07 | 2.54E-07 | 6.658562 |
| ATP6V1G1 | 0.462032 | 5.771919 | 5.376051 | 1.27E-07 | 3.16E-07 | 6.442707 |
| SLC7A11 | 0.586338 | 0.558045 | 5.367394 | 1.32E-07 | 3.29E-07 | 6.399217 |
| FAXDC2 | -0.78816 | 3.218918 | -5.36197 | 1.36E-07 | 3.36E-07 | 6.372002 |
| MFHAS1 | 0.483605 | 1.828056 | 5.357107 | 1.40E-07 | 3.43E-07 | 6.347623 |
| PSMD9 | 0.443677 | 1.493844 | 8.963701 | 1.04E-17 | 6.95E-17 | 29.21097 |
| P3H2 | -0.47148 | 0.652597 | -5.33014 | 1.60E-07 | 3.91E-07 | 6.212793 |
| NDUFS7 | 0.437653 | 3.222412 | 5.328851 | 1.62E-07 | 3.91E-07 | 6.206345 |
| CCND3 | 0.509013 | 2.417255 | 5.266629 | 2.22E-07 | 5.36E-07 | 5.897545 |
| ATP7A | 0.275427 | 0.643792 | 5.252405 | 2.39E-07 | 5.74E-07 | 5.827399 |
| OGFOD1 | 0.335165 | 2.239958 | 5.242425 | 2.51E-07 | 6.01E-07 | 5.77828 |
| CYP2C18 | -1.15339 | 3.800308 | -5.22898 | 2.69E-07 | 6.40E-07 | 5.712255 |
| SMOX | 0.787796 | 1.883858 | 5.216422 | 2.87E-07 | 6.79E-07 | 5.650692 |
| TYW5 | 0.169277 | 0.604413 | 5.207177 | 3.01E-07 | 7.09E-07 | 5.605462 |
| HRG | -1.96476 | 7.512868 | -5.14556 | 4.10E-07 | 9.62E-07 | 5.305809 |
| CAND1 | 0.389586 | 2.33007 | 5.131527 | 4.40E-07 | 1.03E-06 | 5.238015 |
| NDUFV1 | 0.373795 | 5.406385 | 5.128774 | 4.46E-07 | 1.04E-06 | 5.224731 |
| AGPAT4 | 0.362758 | 0.500644 | 5.087731 | 5.47E-07 | 1.27E-06 | 5.027452 |
| ATG4A | 0.356482 | 2.65592 | 5.084691 | 5.56E-07 | 1.28E-06 | 5.0129 |
| GYPC | 0.303276 | 2.238764 | 2.396102 | 0.017007 | 0.023847 | -4.67535 |
| PDZK1IP1 | 1.872462 | 3.221663 | 4.999074 | 8.47E-07 | 1.93E-06 | 4.606104 |
| CYP27B1 | 0.282289 | 0.344792 | 4.97822 | 9.38E-07 | 2.13E-06 | 4.507948 |
| BECN1 | 0.45709 | 2.413078 | 4.884728 | 1.47E-06 | 3.34E-06 | 4.072391 |
| UROD | 0.384395 | 4.814562 | 4.871034 | 1.57E-06 | 3.55E-06 | 4.009209 |
| CYP17A1 | 1.563569 | 2.124325 | 4.865116 | 1.62E-06 | 3.63E-06 | 3.981955 |
| ALKBH1 | 0.239831 | 1.690679 | 4.863092 | 1.64E-06 | 3.65E-06 | 3.972642 |
| HFE | 0.467539 | 2.118782 | 4.832062 | 1.90E-06 | 4.22E-06 | 3.830275 |
| SRI | 0.605704 | 2.636052 | 4.80267 | 2.18E-06 | 4.83E-06 | 3.696172 |
| COX15 | 0.285146 | 3.185922 | 4.759148 | 2.68E-06 | 5.90E-06 | 3.498955 |
| ADI1 | -0.63509 | 5.92799 | -4.75298 | 2.76E-06 | 6.05E-06 | 3.471131 |
| NEK7 | 0.575604 | 2.84858 | 5.331183 | 1.60E-07 | 3.91E-07 | 6.217983 |
| TNRC6B | 0.234092 | 1.079198 | 4.73255 | 3.03E-06 | 6.60E-06 | 3.379216 |
| SLC11A2 | 0.383215 | 2.2362 | 4.627815 | 4.93E-06 | 1.07E-05 | 2.913606 |
| TET1 | 0.164027 | 0.200898 | 4.567727 | 6.49E-06 | 1.40E-05 | 2.650725 |
| SLC22A4 | 0.453938 | 0.610078 | 4.538797 | 7.40E-06 | 1.59E-05 | 2.525268 |
| FN3K | 0.540196 | 4.162394 | 4.536219 | 7.48E-06 | 1.60E-05 | 2.514124 |
| ATP5IF1 | 0.404773 | 5.441197 | 4.520541 | 8.03E-06 | 1.71E-05 | 2.44647 |
| TENT5C | -0.51494 | 1.359214 | -4.51017 | 8.41E-06 | 1.78E-05 | 2.401847 |
| MYL4 | 0.12262 | 0.194035 | 4.504088 | 8.65E-06 | 1.83E-05 | 2.375702 |
| CCDC115 | 0.354281 | 2.473829 | 4.486597 | 9.35E-06 | 1.97E-05 | 2.300726 |
| LPIN2 | -0.63574 | 4.450534 | -4.45446 | 1.08E-05 | 2.26E-05 | 2.16365 |
| DNAJC24 | 0.176276 | 0.646614 | 4.357633 | 1.65E-05 | 3.45E-05 | 1.756115 |
| HBA1 | -0.23944 | 0.213 | -4.35579 | 1.67E-05 | 3.47E-05 | 1.74843 |
| DCAF10 | 0.266586 | 2.178347 | 4.340583 | 1.78E-05 | 3.69E-05 | 1.6852 |
| XK | 0.382104 | 0.632499 | 4.336635 | 1.81E-05 | 3.74E-05 | 1.668817 |
| PHYH | -0.65938 | 6.010403 | -4.33556 | 1.82E-05 | 3.74E-05 | 1.664364 |
| PPEF1 | 0.065943 | 0.067578 | 4.328927 | 1.87E-05 | 3.83E-05 | 1.636864 |
| MELTF | 0.56418 | 0.826316 | 4.269133 | 2.43E-05 | 4.94E-05 | 1.390783 |
| TFR2 | -0.85415 | 6.744492 | -4.23862 | 2.77E-05 | 5.61E-05 | 1.266406 |
| TOP1 | 0.389763 | 3.869226 | 4.208081 | 3.15E-05 | 6.36E-05 | 1.142762 |
| CDR2 | 0.414529 | 2.649155 | 4.196343 | 3.31E-05 | 6.66E-05 | 1.095453 |
| TRAK2 | 0.365999 | 2.183904 | 4.179516 | 3.56E-05 | 7.13E-05 | 1.027843 |
| PC | -0.6271 | 4.999481 | -4.14987 | 4.03E-05 | 8.04E-05 | 0.909344 |
| FTL | 0.661361 | 13.08032 | 4.145447 | 4.10E-05 | 8.16E-05 | 0.891725 |
| SDHB | -0.36004 | 5.337339 | -4.14334 | 4.14E-05 | 8.20E-05 | 0.88336 |
| TCIRG1 | 1.05971 | 4.048591 | 8.50406 | 3.24E-16 | 1.79E-15 | 25.81668 |
| SLC25A38 | 0.322637 | 4.050329 | 4.055186 | 5.97E-05 | 0.000117 | 0.53607 |
| PIGQ | 0.323439 | 2.580751 | 4.051399 | 6.06E-05 | 0.000119 | 0.521307 |
| SLC2A1 | 0.572461 | 1.216754 | 4.005036 | 7.33E-05 | 0.000143 | 0.341593 |
| RANBP10 | 0.338501 | 2.331437 | 3.987462 | 7.87E-05 | 0.000153 | 0.273974 |
| CYP27C1 | 0.142026 | 0.174004 | 3.98265 | 8.03E-05 | 0.000155 | 0.255506 |
| ACO2 | 0.498222 | 4.141583 | 3.981284 | 8.07E-05 | 0.000156 | 0.250269 |
| SIDT2 | 0.278854 | 2.436689 | 3.977881 | 8.19E-05 | 0.000157 | 0.237223 |
| SLC25A28 | 0.301124 | 4.105793 | 3.957379 | 8.89E-05 | 0.00017 | 0.158861 |
| FBXO7 | 0.181548 | 3.694739 | 2.335862 | 0.019968 | 0.027845 | -4.81593 |
| AMBP | -0.84124 | 11.05186 | -3.93172 | 9.86E-05 | 0.000187 | 0.061313 |
| ALOX12 | 0.186301 | 0.311721 | 6.142985 | 1.88E-09 | 5.47E-09 | 10.53277 |
| NEO1 | 0.418053 | 1.981588 | 3.924412 | 0.000102 | 0.000191 | 0.033645 |
| MYC | -0.79781 | 3.697715 | -3.91008 | 0.000108 | 0.000202 | -0.02051 |
| CYP4A11 | -2.48235 | 5.622202 | -9.13416 | 2.81E-18 | 1.96E-17 | 30.50064 |
| CYP1A1 | -1.21191 | 1.934293 | -3.88506 | 0.000119 | 0.000222 | -0.11457 |
| DCAF11 | -0.40023 | 4.36383 | -3.88061 | 0.000121 | 0.000225 | -0.13123 |
| LAMP2 | 0.431965 | 5.597172 | 3.865265 | 0.000129 | 0.000238 | -0.18857 |
| FTO | 0.243179 | 1.446797 | 3.863068 | 0.00013 | 0.000239 | -0.19676 |
| TH | -0.09455 | 0.056566 | -3.8589 | 0.000132 | 0.000242 | -0.2123 |
| DPYD | -0.53928 | 2.813807 | -3.85498 | 0.000134 | 0.000245 | -0.22688 |
| CDO1 | -0.91693 | 5.751801 | -3.84158 | 0.000141 | 0.000257 | -0.27669 |
| CYP4F12 | -0.5787 | 2.538956 | -3.84119 | 0.000141 | 0.000257 | -0.27814 |
| EPB41 | 0.42669 | 2.409944 | 3.827763 | 0.000149 | 0.00027 | -0.32784 |
| ABCG2 | -0.67136 | 2.166787 | -3.82135 | 0.000153 | 0.000275 | -0.35153 |
| C3 | -0.64816 | 10.01647 | -3.82112 | 0.000153 | 0.000275 | -0.35237 |
| MIOX | 0.412403 | 0.399029 | 3.815412 | 0.000156 | 0.00028 | -0.37343 |
| CYP4F3 | -0.6841 | 4.640669 | -3.81127 | 0.000159 | 0.000284 | -0.38867 |
| TSPAN5 | 0.493437 | 0.675981 | 3.809303 | 0.00016 | 0.000285 | -0.39593 |
| SC5D | -0.56092 | 4.031167 | -3.78884 | 0.000173 | 0.000308 | -0.47105 |
| SFXN1 | -0.32127 | 3.523629 | -3.7767 | 0.000182 | 0.000321 | -0.51543 |
| AGMO | -0.58719 | 3.770121 | -3.76228 | 0.000192 | 0.000339 | -0.56797 |
| LMO2 | 0.333301 | 1.833734 | 3.753101 | 0.000199 | 0.00035 | -0.60133 |
| CYP27A1 | -0.71914 | 7.10038 | -3.74902 | 0.000202 | 0.000354 | -0.61614 |
| ARL2BP | 0.31547 | 1.164887 | 3.744429 | 0.000206 | 0.000359 | -0.63277 |
| NUBP1 | 0.246214 | 2.916271 | 3.717662 | 0.000228 | 0.000396 | -0.72938 |
| CYP21A2 | 0.503387 | 1.483758 | 3.716872 | 0.000229 | 0.000396 | -0.73222 |
| CYP7A1 | 1.222226 | 3.181901 | 3.715268 | 0.00023 | 0.000398 | -0.73799 |
| TF | -0.93219 | 9.144861 | -3.68612 | 0.000258 | 0.000443 | -0.84239 |
| SMAD4 | 0.244549 | 2.125768 | 3.680939 | 0.000263 | 0.00045 | -0.86087 |
| RAP1GAP | 0.678804 | 2.673637 | 3.606043 | 0.000348 | 0.000595 | -1.12526 |
| LTF | 0.232363 | 0.484212 | 2.175296 | 0.030164 | 0.041273 | -5.17358 |
| SLC30A10 | 0.510894 | 2.684344 | 3.592497 | 0.000366 | 0.000621 | -1.17252 |
| GDE1 | 0.272436 | 3.022071 | 3.591205 | 0.000368 | 0.000622 | -1.17703 |
| COX10 | 0.278715 | 2.021955 | 3.572012 | 0.000395 | 0.000666 | -1.24369 |
| PAH | -0.75218 | 6.151031 | -3.49658 | 0.000521 | 0.000876 | -1.50245 |
| SLC30A1 | -0.46719 | 4.065554 | -3.46712 | 0.00058 | 0.000972 | -1.60209 |
| SYNJ1 | 0.18088 | 0.868996 | 3.461349 | 0.000592 | 0.000989 | -1.62152 |
| UQCRFS1 | 0.274159 | 4.357735 | 3.419483 | 0.000689 | 0.001146 | -1.76154 |
| ATP6V0D2 | 0.280598 | 0.298292 | 3.384506 | 0.00078 | 0.001293 | -1.87728 |
| ACSL6 | 0.37556 | 0.650561 | 3.367219 | 0.000829 | 0.001367 | -1.93406 |
| RIOK3 | 0.257561 | 3.488356 | 3.367059 | 0.00083 | 0.001367 | -1.93459 |
| RHD | 0.055922 | 0.131238 | 3.337495 | 0.000921 | 0.001512 | -2.03106 |
| ABCB7 | 0.235064 | 2.724284 | 3.30215 | 0.001041 | 0.001704 | -2.14534 |
| STEAP2 | 0.382307 | 0.859962 | 3.268643 | 0.001169 | 0.001908 | -2.25261 |
| ATP6V1B1 | 0.192147 | 0.221367 | 3.250983 | 0.001243 | 0.002021 | -2.30873 |
| ALDH1L1 | -0.91029 | 4.390118 | -3.24522 | 0.001267 | 0.002055 | -2.32699 |
| XPO7 | 0.294827 | 2.339319 | 3.230333 | 0.001334 | 0.002155 | -2.37398 |
| RNF123 | 0.292231 | 2.288212 | 3.210935 | 0.001425 | 0.002295 | -2.43492 |
| ATP6V1G2 | 0.106797 | 0.239686 | 3.195592 | 0.001501 | 0.00241 | -2.48287 |
| ALKBH8 | 0.144701 | 0.984562 | 3.172395 | 0.001623 | 0.002597 | -2.55495 |
| CYP2W1 | 0.157181 | 0.159737 | 3.146267 | 0.001771 | 0.002826 | -2.63554 |
| C1QA | -0.61324 | 5.947144 | -3.06365 | 0.002327 | 0.003702 | -2.88618 |
| MIR210 | 0.196043 | 0.297082 | 3.054906 | 0.002395 | 0.003797 | -2.91232 |
| SEC14L1 | 0.252827 | 1.984305 | 3.04379 | 0.002483 | 0.003925 | -2.94547 |
| TFAP2A | 0.209537 | 0.238162 | 3.035194 | 0.002553 | 0.004023 | -2.97102 |
| CAST | 0.236339 | 3.387164 | 3.010482 | 0.002765 | 0.004345 | -3.04409 |
| HEPHL1 | 0.031134 | 0.056937 | 2.981654 | 0.003034 | 0.004751 | -3.12861 |
| SPTB | 0.074992 | 0.114673 | 2.941428 | 0.003448 | 0.005383 | -3.24525 |
| CYP1B1 | 0.497858 | 1.60613 | 2.911938 | 0.003783 | 0.005889 | -3.32979 |
| PHF2 | 0.265459 | 2.058005 | 2.908638 | 0.003823 | 0.005932 | -3.3392 |
| KDM7A | -0.20222 | 1.317712 | -2.90414 | 0.003877 | 0.005998 | -3.352 |
| RNF19A | 0.341326 | 3.093923 | 2.892114 | 0.004025 | 0.006209 | -3.38616 |
| DCUN1D1 | 0.197604 | 2.056918 | 2.884885 | 0.004117 | 0.006331 | -3.40662 |
| ATP6V0E2 | 0.487398 | 4.416012 | 2.868648 | 0.00433 | 0.006638 | -3.4524 |
| TMCC2 | 0.112078 | 0.212658 | 2.844152 | 0.00467 | 0.007139 | -3.52101 |
| CISD3 | 0.266998 | 5.003495 | 2.832914 | 0.004834 | 0.007368 | -3.55229 |
| GLRX5 | 0.228904 | 4.535502 | 2.795909 | 0.005413 | 0.008224 | -3.65445 |
| P4HTM | 0.294292 | 1.655022 | 2.785878 | 0.00558 | 0.008453 | -3.68193 |
| HEBP1 | -0.24193 | 4.076756 | -2.76371 | 0.005966 | 0.009011 | -3.7423 |
| MDM2 | 0.249056 | 2.588402 | 2.744463 | 0.006321 | 0.009518 | -3.79434 |
| FLVCR1 | 1.211923 | 1.47831 | 12.41983 | 2.18E-30 | 6.18E-29 | 58.11146 |
| CTSB | -0.33718 | 6.883699 | -2.72979 | 0.006604 | 0.009886 | -3.83379 |
| ACKR1 | 0.332734 | 0.716042 | 2.70307 | 0.007149 | 0.010671 | -3.90507 |
| EPAS1 | -0.27339 | 4.628894 | -2.67351 | 0.007798 | 0.011606 | -3.98314 |
| ALOX15B | 0.399522 | 0.478136 | 2.645616 | 0.00846 | 0.012554 | -4.05607 |
| PRDX2 | 0.552507 | 6.362157 | 6.622381 | 1.08E-10 | 3.51E-10 | 13.3199 |
| IREB2 | 0.194522 | 1.977733 | 2.604216 | 0.009535 | 0.014023 | -4.16293 |
| ALKBH3 | 0.23616 | 2.305402 | 2.603321 | 0.009559 | 0.014023 | -4.16523 |
| TPH1 | 0.065442 | 0.073674 | 2.603301 | 0.00956 | 0.014023 | -4.16528 |
| EPOR | 0.213044 | 1.501609 | 2.572113 | 0.01045 | 0.015285 | -4.24468 |
| TYR | 0.019016 | 0.017237 | 1.256217 | 0.209735 | 0.249428 | -6.73798 |
| REP15 | 0.115607 | 0.281925 | 2.558885 | 0.01085 | 0.015779 | -4.27807 |
| PLOD2 | 0.355911 | 3.279413 | 2.537424 | 0.011527 | 0.016699 | -4.3319 |
| CLCN3 | 0.247785 | 2.435869 | 2.535913 | 0.011577 | 0.016699 | -4.33567 |
| ABCE1 | 0.200809 | 2.681548 | 2.535767 | 0.011581 | 0.016699 | -4.33604 |
| ACO1 | -0.26415 | 4.195363 | -2.5316 | 0.011718 | 0.016848 | -4.34644 |
| CYP4X1 | -0.28465 | 1.093287 | -2.52078 | 0.012078 | 0.017318 | -4.37333 |
| CYP7B1 | -0.32158 | 1.886265 | -2.51058 | 0.012428 | 0.017769 | -4.39861 |
| CYBRD1 | -0.37818 | 2.087517 | -2.4937 | 0.013025 | 0.018543 | -4.44019 |
| NNT | -0.32448 | 3.884622 | -2.49324 | 0.013042 | 0.018543 | -4.44134 |
| PDX1 | 0.419555 | 0.754225 | 2.489334 | 0.013184 | 0.018693 | -4.45092 |
| ATP6V0A1 | 0.742665 | 2.886127 | 9.452618 | 2.35E-19 | 1.89E-18 | 32.95315 |
| TBXAS1 | -0.20433 | 0.995186 | -2.45742 | 0.014397 | 0.020299 | -4.52867 |
| CCDC28A | -0.19906 | 3.930798 | -2.41588 | 0.016123 | 0.02267 | -4.62843 |
| MXI1 | -0.11204 | 3.060753 | -1.27176 | 0.204162 | 0.243369 | -6.71842 |
| CYP11B2 | 0.038151 | 0.044483 | 0.612055 | 0.540832 | 0.581995 | -7.33803 |
| DNM2 | 0.721536 | 2.942723 | 8.949689 | 1.15E-17 | 7.63E-17 | 29.10568 |
| DMTN | 0.327429 | 2.480749 | 2.320928 | 0.020768 | 0.028883 | -4.85025 |
| CYP3A7 | -0.76184 | 3.595714 | -2.28717 | 0.022682 | 0.031446 | -4.92702 |
| ELP3 | 0.173744 | 2.448715 | 2.286276 | 0.022735 | 0.031446 | -4.92903 |
| MPP1 | 0.263944 | 2.646474 | 2.264633 | 0.024044 | 0.033166 | -4.97766 |
| DNA2 | 0.548977 | 0.741718 | 8.037564 | 9.37E-15 | 4.41E-14 | 22.50172 |
| SOD1 | -0.25736 | 7.959111 | -2.19949 | 0.028387 | 0.038945 | -5.12128 |
| RTEL1 | 0.090657 | 0.138536 | 6.111079 | 2.26E-09 | 6.50E-09 | 10.35344 |
| ALAS2 | -0.08283 | 0.099345 | -2.171 | 0.03049 | 0.041607 | -5.18281 |
| RSAD2 | -0.20596 | 0.911552 | -1.80663 | 0.071535 | 0.09265 | -5.90031 |
| CYP19A1 | 0.170736 | 0.15323 | 2.105916 | 0.035804 | 0.048598 | -5.32042 |
| CYP4Z2P | 0.01491 | 0.018731 | 2.097115 | 0.03658 | 0.049445 | -5.33872 |
| CYP2F1 | 0.009811 | 0.011184 | 2.096645 | 0.036622 | 0.049445 | -5.33969 |
| MSMO1 | -0.31594 | 6.100369 | -2.06479 | 0.039556 | 0.053264 | -5.40527 |
| ARHGEF12 | -0.19042 | 2.655619 | -2.05364 | 0.040628 | 0.054564 | -5.42798 |
| HAGH | -0.26174 | 4.555659 | -2.05155 | 0.040832 | 0.054694 | -5.43222 |
| KAT2B | -0.22608 | 2.550872 | -2.02542 | 0.043456 | 0.058055 | -5.48494 |
| FTH1P19 | 0.044391 | 0.050476 | 2.021776 | 0.043832 | 0.058405 | -5.49224 |
| BLVRB | 0.237617 | 6.345929 | 1.999872 | 0.046157 | 0.061342 | -5.53585 |
| FOXO3 | 0.18517 | 2.294903 | 1.993446 | 0.046858 | 0.062112 | -5.54855 |
| FDX1 | -0.172 | 3.803136 | -1.99205 | 0.047012 | 0.062153 | -5.5513 |
| CYP2D7 | -0.41095 | 2.450591 | -1.97651 | 0.04875 | 0.064285 | -5.58184 |
| HBD | -0.10251 | 0.102299 | -1.96043 | 0.050605 | 0.066558 | -5.61318 |
| CYP2U1 | -0.13155 | 1.026484 | -1.91113 | 0.056668 | 0.07434 | -5.70774 |
| CROCCP2 | 0.174947 | 2.431077 | 1.90635 | 0.057286 | 0.074958 | -5.71677 |
| BCL2 | 0.130289 | 0.693302 | 1.88411 | 0.06024 | 0.078621 | -5.75854 |
| CH25H | -0.24152 | 0.782827 | -1.85143 | 0.064809 | 0.084367 | -5.81905 |
| REV3L | 0.103682 | 0.883253 | 1.832922 | 0.067521 | 0.087674 | -5.85285 |
| RBM38 | 0.540802 | 2.800295 | 4.746604 | 2.84E-06 | 6.21E-06 | 3.442407 |
| KLF1 | 0.037325 | 0.068215 | 1.741386 | 0.082347 | 0.106383 | -6.01513 |
| NTHL1 | 0.198779 | 3.554744 | 1.735741 | 0.083342 | 0.107395 | -6.02487 |
| CYP26C1 | 0.008469 | 0.016059 | 1.723899 | 0.085461 | 0.109848 | -6.0452 |
| HMOX2 | 0.155335 | 3.876153 | 1.713356 | 0.087384 | 0.112037 | -6.06318 |
| CTSE | 0.242215 | 0.386525 | 1.687725 | 0.092206 | 0.117921 | -6.10645 |
| CYP26B1 | 0.174819 | 0.435856 | 1.685477 | 0.092639 | 0.118178 | -6.11021 |
| TRIM10 | 0.128475 | 0.846289 | 1.637822 | 0.102207 | 0.130058 | -6.18884 |
| SPTA1 | 0.0812 | 0.082445 | 1.605662 | 0.109099 | 0.138482 | -6.24065 |
| FBXL5 | 0.252725 | 3.885014 | 2.567243 | 0.010596 | 0.015454 | -4.25699 |
| CCND1 | -0.28881 | 4.345495 | -1.55909 | 0.119727 | 0.151218 | -6.31386 |
| ANK1 | 0.051641 | 0.075715 | 1.537641 | 0.124889 | 0.157347 | -6.34687 |
| STEAP1 | 0.333217 | 2.744405 | 1.532498 | 0.126152 | 0.158546 | -6.35472 |
| FA2H | 0.170864 | 0.309518 | 1.508872 | 0.132083 | 0.165585 | -6.39043 |
| ADD2 | 0.034967 | 0.060688 | 1.507618 | 0.132403 | 0.165585 | -6.39231 |
| CYP4F22 | 0.363048 | 2.166159 | 1.504371 | 0.133237 | 0.166219 | -6.39718 |
| ATP6V1G3 | 0.017938 | 0.015807 | 1.489275 | 0.137165 | 0.170701 | -6.41964 |
| TSPO | 0.696776 | 4.636611 | 4.059622 | 5.86E-05 | 0.000116 | 0.553381 |
| FTHL17 | 0.090904 | 0.080107 | 1.455172 | 0.146368 | 0.181268 | -6.46957 |
| ISCA1 | 0.271377 | 3.300824 | 3.902486 | 0.000111 | 0.000207 | -0.0491 |
| KEL | 0.168726 | 0.29933 | 1.43559 | 0.151863 | 0.187162 | -6.49772 |
| NUBPL | 0.107014 | 1.205581 | 1.431166 | 0.153125 | 0.188263 | -6.50402 |
| BMP6 | -0.13879 | 0.543417 | -1.41728 | 0.157142 | 0.192735 | -6.5237 |
| NDUFV2 | 0.120173 | 3.02109 | 1.413191 | 0.15834 | 0.193738 | -6.52945 |
| MAP2K3 | -0.5802 | 3.870299 | -6.41064 | 3.90E-10 | 1.21E-09 | 12.06765 |
| TTYH1 | 0.136809 | 0.275907 | 1.387013 | 0.166173 | 0.202349 | -6.56592 |
| TNS1 | 0.157989 | 2.57535 | 1.382118 | 0.167669 | 0.203684 | -6.57266 |
| ETHE1 | -0.13084 | 3.861249 | -1.35611 | 0.175793 | 0.213045 | -6.6081 |
| CYP24A1 | 0.0376 | 0.037539 | 1.350746 | 0.177503 | 0.214606 | -6.61532 |
| TET2 | 0.054248 | 0.512274 | 1.342176 | 0.180263 | 0.217426 | -6.6268 |
| ALOX12B | 0.015115 | 0.02691 | 1.33541 | 0.182465 | 0.219561 | -6.63582 |
| NUBP2 | 0.579584 | 3.482752 | 8.284461 | 1.60E-15 | 8.42E-15 | 24.23945 |
| CYP20A1 | 0.180674 | 1.55546 | 3.947466 | 9.26E-05 | 0.000176 | 0.121105 |
| ERCC2 | 0.828181 | 2.272952 | 10.92314 | 1.28E-24 | 1.81E-23 | 44.94508 |
| CYP46A1 | 0.01001 | 0.101689 | 0.732192 | 0.464459 | 0.512819 | -7.2574 |
| PICALM | 0.215581 | 4.19837 | 2.626335 | 0.008946 | 0.013237 | -4.10604 |
| E2F2 | 0.501067 | 0.50102 | 7.636801 | 1.51E-13 | 6.27E-13 | 19.76387 |
| STEAP3 | -1.5127 | 4.235697 | -8.64547 | 1.14E-16 | 6.89E-16 | 26.84774 |
| HEPH | 0.087924 | 0.620572 | 1.224043 | 0.221622 | 0.261729 | -6.77771 |
| HBQ1 | 0.060908 | 0.082365 | 1.210791 | 0.226656 | 0.267055 | -6.79378 |
| CYP51A1 | 0.293654 | 1.615156 | 2.468909 | 0.013949 | 0.019723 | -4.5008 |
| TMLHE | 0.070332 | 1.584651 | 1.189981 | 0.234725 | 0.275288 | -6.81866 |
| CYP4F8 | 0.008488 | 0.008418 | 1.16409 | 0.245048 | 0.286563 | -6.84902 |
| SCD5 | -0.15623 | 0.680157 | -1.16306 | 0.245464 | 0.286563 | -6.85022 |
| ELL2 | -0.14212 | 3.867544 | -1.15802 | 0.247513 | 0.288293 | -6.85604 |
| CYP2S1 | 0.130856 | 0.767989 | 1.110354 | 0.267481 | 0.31084 | -6.90991 |
| TYW1B | 0.083335 | 0.84524 | 1.050996 | 0.293864 | 0.340001 | -6.97385 |
| GCLC | -0.12247 | 3.457604 | -1.0509 | 0.29391 | 0.340001 | -6.97396 |
| PHF8 | 0.255942 | 2.212233 | 2.73018 | 0.006596 | 0.009886 | -3.83273 |
| NUDT4 | 0.257936 | 1.645901 | 3.602912 | 0.000352 | 0.0006 | -1.13619 |
| ALOXE3 | 0.015243 | 0.025152 | 1.007846 | 0.314108 | 0.360905 | -7.01814 |
| NCOA4 | -0.10267 | 5.794336 | -1.00008 | 0.317849 | 0.364381 | -7.02592 |
| AHSP | -0.05698 | 0.11401 | -0.96794 | 0.33363 | 0.381613 | -7.05745 |
| IFNG | 0.05354 | 0.213589 | 0.94518 | 0.34511 | 0.393858 | -7.07916 |
| BTRC | 0.374641 | 1.529194 | 6.371965 | 4.91E-10 | 1.51E-09 | 11.84253 |
| SLC6A8 | 1.320953 | 1.704253 | 6.274505 | 8.73E-10 | 2.61E-09 | 11.2802 |
| ATP6V1B2 | 0.075295 | 2.731335 | 0.884043 | 0.377178 | 0.427581 | -7.13494 |
| CYP11A1 | 0.126118 | 1.301736 | 0.868668 | 0.385524 | 0.436071 | -7.14839 |
| CYP3A5 | -0.18547 | 4.59869 | -0.85165 | 0.394892 | 0.445676 | -7.16299 |
| CLIC2 | -0.0221 | 1.482972 | -0.19951 | 0.841957 | 0.864025 | -7.50527 |
| UBB | 0.064456 | 8.482919 | 0.847592 | 0.397148 | 0.446243 | -7.16643 |
| TMPRSS6 | -0.19094 | 5.102437 | -0.8435 | 0.399429 | 0.447818 | -7.16989 |
| GCLM | 0.114506 | 3.321371 | 0.839955 | 0.401411 | 0.449051 | -7.17286 |
| SLC25A37 | -0.06279 | 1.499019 | -0.81031 | 0.418223 | 0.4664 | -7.19727 |
| CYP2A13 | -0.13786 | 0.744946 | -0.80938 | 0.418752 | 0.4664 | -7.19802 |
| DRD2 | 0.01066 | 0.011854 | 0.783189 | 0.433957 | 0.48228 | -7.21884 |
| P4HA1 | -0.13155 | 4.131611 | -0.7702 | 0.441616 | 0.489722 | -7.22891 |
| NFE2 | 0.045809 | 0.208515 | 0.737883 | 0.460997 | 0.510103 | -7.25323 |
| P3H3 | 0.163548 | 0.832009 | 1.462644 | 0.144312 | 0.179158 | -6.45873 |
| TAL1 | -0.02461 | 0.328479 | -0.69132 | 0.489744 | 0.539566 | -7.28644 |
| ALAD | -0.29112 | 4.99167 | -2.16841 | 0.030688 | 0.041765 | -5.18837 |
| ACP5 | 0.140334 | 3.370008 | 0.674623 | 0.500286 | 0.548805 | -7.29783 |
| TRIM58 | -0.00801 | 0.030261 | -0.67159 | 0.502214 | 0.549736 | -7.29987 |
| SELENBP1 | -0.11421 | 5.690124 | -0.65907 | 0.51021 | 0.557289 | -7.30819 |
| MAP1LC3A | -0.09943 | 3.573347 | -0.64959 | 0.516311 | 0.562745 | -7.31438 |
| CYP2D6 | -0.17701 | 5.568761 | -0.64704 | 0.517957 | 0.563334 | -7.31603 |
| CYP4F11 | -0.12537 | 4.239593 | -0.64414 | 0.519833 | 0.564168 | -7.3179 |
| TFF1 | 0.13405 | 0.624921 | 0.63761 | 0.524075 | 0.567562 | -7.32208 |
| GATA1 | 0.011398 | 0.045979 | 0.632961 | 0.527103 | 0.569629 | -7.32503 |
| HBBP1 | 0.007006 | 0.008408 | 0.618201 | 0.536778 | 0.578856 | -7.33425 |
| EGLN3 | 0.52713 | 0.846959 | 3.926622 | 0.000101 | 0.00019 | 0.042008 |
| GYPE | 0.008985 | 0.052399 | 0.605576 | 0.545123 | 0.585375 | -7.34197 |
| GYPA | 0.001036 | 0.01277 | 0.0812 | 0.935322 | 0.950116 | -7.52186 |
| HBZ | 0.002771 | 0.004826 | 0.566883 | 0.571096 | 0.610689 | -7.36462 |
| MOCS1 | 0.057498 | 3.151912 | 0.555223 | 0.579037 | 0.617883 | -7.37115 |
| ERMAP | 0.041758 | 1.841075 | 0.549104 | 0.583226 | 0.62105 | -7.37453 |
| CYP11B1 | 0.004226 | 0.003933 | 0.543776 | 0.586884 | 0.623641 | -7.37743 |
| NR3C1 | 0.042957 | 2.773096 | 0.526992 | 0.598477 | 0.634635 | -7.38641 |
| DAAM1 | 0.046477 | 1.498465 | 0.518493 | 0.604387 | 0.639569 | -7.39085 |
| RHAG | 0.013913 | 0.031312 | 0.49775 | 0.618921 | 0.65359 | -7.40138 |
| CA1 | 0.021674 | 0.086973 | 0.423633 | 0.67205 | 0.708227 | -7.43549 |
| MINPP1 | 0.03981 | 3.18062 | 0.401087 | 0.68856 | 0.724126 | -7.44478 |
| CYGB | -0.05267 | 2.393596 | -0.38742 | 0.698638 | 0.73321 | -7.45016 |
| PPEF2 | -0.00125 | 0.003953 | -0.37514 | 0.707746 | 0.74124 | -7.45484 |
| P4HA3 | -0.01547 | 0.283233 | -0.36792 | 0.713119 | 0.74466 | -7.45752 |
| BNIP3L | -0.0388 | 2.955314 | -0.36682 | 0.713938 | 0.74466 | -7.45792 |
| EPB42 | 0.00385 | 0.018394 | 0.303693 | 0.761512 | 0.792658 | -7.47907 |
| BMP2K | -0.01078 | 0.44606 | -0.29129 | 0.770977 | 0.800872 | -7.48276 |
| BACH1 | 0.02789 | 2.219039 | 0.282675 | 0.777565 | 0.80607 | -7.48523 |
| UQCRFS1P1 | 0.010635 | 0.221009 | 0.256284 | 0.797857 | 0.825425 | -7.49234 |
| MOCOS | 0.024242 | 2.502636 | 0.243103 | 0.808044 | 0.832962 | -7.49562 |
| SNCA | -0.01076 | 0.168242 | -0.24262 | 0.808415 | 0.832962 | -7.49574 |
| NDUFS1 | -0.01561 | 3.21081 | -0.22604 | 0.821284 | 0.844512 | -7.49963 |
| FBXO34 | 0.171772 | 1.949722 | 2.234594 | 0.025969 | 0.035725 | -5.04439 |
| TPH2 | 0.000802 | 0.005748 | 0.196629 | 0.844213 | 0.864596 | -7.50584 |
| CYP2G1P | 0.003383 | 0.067787 | 0.143178 | 0.886218 | 0.905793 | -7.51491 |
| HJV | -0.03356 | 5.687135 | -0.12395 | 0.901411 | 0.919476 | -7.51748 |
| CPOX | -0.00676 | 2.56497 | -0.08123 | 0.935294 | 0.950116 | -7.52186 |
| RAD23A | 0.573426 | 4.968207 | 7.98058 | 1.40E-14 | 6.42E-14 | 22.10611 |
| RHCE | 0.004704 | 0.451082 | 0.079026 | 0.937049 | 0.950116 | -7.52204 |
| FLVCR2 | -0.00763 | 1.890161 | -0.07568 | 0.939711 | 0.95092 | -7.5223 |
| FECH | 0.005427 | 2.689629 | 0.063149 | 0.949678 | 0.959099 | -7.52317 |
| BTG2 | 0.009186 | 3.332534 | 0.055618 | 0.955673 | 0.963242 | -7.52361 |
| KLF3 | -0.00393 | 3.225388 | -0.04611 | 0.963242 | 0.968953 | -7.5241 |
| GYPB | -0.00019 | 0.005736 | -0.02006 | 0.984003 | 0.987885 | -7.52496 |
| UCP2 | 0.00241 | 2.890399 | 0.015639 | 0.98753 | 0.989474 | -7.52504 |
| ICAM4 | 0 | 0 | 0 | 1 | 1 | -7.52516 |
