# Supplementary material for: A novel four-gene of iron metabolism-related and methylated for prognosis prediction of hepatocellular carcinoma
Source: Bioengineered. 2020 Dec 31;12(1):240–51. doi: 10.1080/21655979.2020.1866303 (PMC8806199; doi:10.1080/21655979.2020.1866303)
Supplement: Supplemental Material [file KBIE_A_1866303_SM2202.zip › supplement/Supplementary Table4.docx]

**Table S4:** Methylation status of differentially expressed iron metabolism-related genes.

| lnc | m_gene | cor | p.adj | gene | chrom | chromStart | chromEnd | strand |
| --- | --- | --- | --- | --- | --- | --- | --- | --- |
| cg00046021 | PLOD3 | -0.35544079 | 1.03E-13 | PLOD3 | chr7 | 101215170 | 101215171 | . |
| cg00370022 | CYP1A1 | -0.323218184 | 1.79E-11 | CYP1A1 | chr15 | 74722851 | 74722852 | . |
| cg00460795 | STEAP3 | -0.477412264 | 7.64E-25 | STEAP3 | chr2 | 119240496 | 119240497 | . |
| cg00477978 | HPX | -0.46851631 | 7.19E-24 | HPX | chr11 | 6442516 | 6442517 | . |
| cg00506866 | RRM2 | -0.421995317 | 3.20E-19 | RRM2 | chr2 | 10123353 | 10123354 | . |
| cg00590251 | STEAP3 | -0.616549355 | 1.68E-44 | STEAP3 | chr2 | 119223007 | 119223008 | . |
| cg01032570 | SLC39A14 | -0.42745547 | 1.22E-19 | SLC39A14 | chr8 | 22367021 | 22367022 | . |
| cg01217071 | AOX1 | -0.324197436 | 1.55E-11 | AOX1 | chr2 | 200585600 | 200585601 | . |
| cg02291556 | PDZK1IP1 | -0.430920551 | 4.66E-20 | PDZK1IP1 | chr1 | 47190468 | 47190469 | . |
| cg03389720 | ABAT | -0.327092798 | 9.95E-12 | ABAT | chr16 | 8686191 | 8686192 | . |
| cg04413147 | FTCD | -0.461059907 | 4.47E-23 | FTCD | chr21 | 46155220 | 46155221 | . |
| cg04767697 | HAAO | -0.338823829 | 1.69E-12 | HAAO | chr2 | 42789306 | 42789307 | . |
| cg04968473 | CYP1A2 | -0.544907289 | 3.10E-33 | CYP1A2 | chr15 | 74748393 | 74748394 | . |
| cg05194426 | CYP2E1 | -0.410198489 | 3.75E-18 | CYP2E1 | chr10 | 133529689 | 133529690 | . |
| cg05209330 | P4HA2 | -0.357560737 | 7.18E-14 | P4HA2 | chr5 | 132215249 | 132215250 | . |
| cg05858136 | ALKBH2 | -0.419695075 | 5.21E-19 | ALKBH2 | chr12 | 109092275 | 109092276 | . |
| cg05885577 | CYP17A1 | -0.336454009 | 2.32E-12 | CYP17A1 | chr10 | 102837602 | 102837603 | . |
| cg06619077 | PDZK1IP1 | -0.394891439 | 1.03E-16 | PDZK1IP1 | chr1 | 47190331 | 47190332 | . |
| cg07150145 | PDZK1IP1 | -0.480686866 | 3.30E-25 | PDZK1IP1 | chr1 | 47190465 | 47190466 | . |
| cg08350126 | HPX | -0.520512493 | 5.54E-30 | HPX | chr11 | 6442246 | 6442247 | . |
| cg09201719 | CYP17A1 | -0.582603996 | 8.16E-39 | CYP17A1 | chr10 | 102837133 | 102837134 | . |
| cg09258479 | PDZK1IP1 | -0.391741586 | 1.46E-16 | PDZK1IP1 | chr1 | 47190189 | 47190190 | . |
| cg09436823 | FTCD | -0.334188248 | 3.31E-12 | FTCD | chr21 | 46155584 | 46155585 | . |
| cg09441069 | FTCD | -0.374196271 | 3.85E-15 | FTCD | chr21 | 46149565 | 46149566 | . |
| cg09457255 | CP | -0.358810713 | 5.80E-14 | CP | chr3 | 149216054 | 149216055 | . |
| cg09550024 | CYP2C9 | -0.377158676 | 2.25E-15 | CYP2C9 | chr10 | 94938609 | 94938610 | . |
| cg10394047 | FTCD | -0.417913629 | 7.59E-19 | FTCD | chr21 | 46155502 | 46155503 | . |
| cg10985065 | CYP2E1 | -0.372385962 | 5.34E-15 | CYP2E1 | chr10 | 133525131 | 133525132 | . |
| cg11445109 | CYP2E1 | -0.341468388 | 1.04E-12 | CYP2E1 | chr10 | 133529744 | 133529745 | . |
| cg12651712 | CYP4A11 | -0.426128528 | 1.32E-19 | CYP4A11 | chr1 | 46941371 | 46941372 | . |
| cg13656062 | CYP4F2 | -0.493635014 | 1.08E-26 | CYP4F2 | chr19 | 15897913 | 15897914 | . |
| cg14196507 | CYP2C18 | -0.465074911 | 1.68E-23 | CYP2C18 | chr10 | 94683626 | 94683627 | . |
| cg14473416 | AQP3 | -0.417288749 | 8.65E-19 | AQP3 | chr9 | 33443367 | 33443368 | . |
| cg14527110 | P4HA2 | -0.447731336 | 1.05E-21 | P4HA2 | chr5 | 132225599 | 132225600 | . |
| cg15187606 | PDZK1IP1 | -0.408906118 | 4.89E-18 | PDZK1IP1 | chr1 | 47191181 | 47191182 | . |
| cg15257755 | SLC39A14 | -0.318901271 | 3.42E-11 | SLC39A14 | chr8 | 22368155 | 22368156 | . |
| cg17489534 | BCAM | -0.367990637 | 1.17E-14 | BCAM | chr19 | 44812315 | 44812316 | . |
| cg17886959 | MT2A | -0.300190672 | 5.01E-10 | MT2A | chr16 | 56608112 | 56608113 | . |
| cg18024037 | FTCD | -0.341481954 | 1.04E-12 | FTCD | chr21 | 46155590 | 46155591 | . |
| cg18623836 | RRM2 | -0.383990503 | 6.35E-16 | RRM2 | chr2 | 10121780 | 10121781 | . |
| cg19046783 | CYP3A4 | -0.687815336 | 5.16E-59 | CYP3A4 | chr7 | 99784463 | 99784464 | . |
| cg19516340 | RRM2 | -0.363265115 | 2.68E-14 | RRM2 | chr2 | 10123569 | 10123570 | . |
| cg19725553 | BLVRA | -0.344699429 | 6.15E-13 | BLVRA | chr7 | 43758299 | 43758300 | . |
| cg20251008 | PPOX | -0.315585284 | 5.58E-11 | PPOX | chr1 | 161166255 | 161166256 | . |
| cg20910746 | BBOX1 | -0.308072277 | 1.66E-10 | BBOX1 | chr11 | 27040869 | 27040870 | . |
| cg20994950 | PPOX | -0.301611343 | 4.12E-10 | PPOX | chr1 | 161168307 | 161168308 | . |
| cg23318764 | BCAM | -0.40687474 | 7.38E-18 | BCAM | chr19 | 44812464 | 44812465 | . |
| cg23420841 | ATP6V1C1 | -0.479820193 | 4.12E-25 | ATP6V1C1 | chr8 | 103023770 | 103023771 | . |
| cg24117468 | P4HA2 | -0.309493728 | 1.35E-10 | P4HA2 | chr5 | 132227155 | 132227156 | . |
| cg24140586 | CYP2C9 | -0.515521558 | 2.38E-29 | CYP2C9 | chr10 | 94937679 | 94937680 | . |
| cg24483002 | SLC39A14 | -0.370152365 | 7.97E-15 | SLC39A14 | chr8 | 22367504 | 22367505 | . |
| cg25322086 | FTCD | -0.316211266 | 5.09E-11 | FTCD | chr21 | 46155633 | 46155634 | . |
| cg25677697 | POLD1 | -0.311084167 | 1.07E-10 | POLD1 | chr19 | 50409092 | 50409093 | . |
| cg26523175 | PDZK1IP1 | -0.426967456 | 1.10E-19 | PDZK1IP1 | chr1 | 47190751 | 47190752 | . |
| cg26651122 | MT2A | -0.316918207 | 4.59E-11 | MT2A | chr16 | 56607865 | 56607866 | . |
| cg26888222 | STEAP3 | -0.548979723 | 8.37E-34 | STEAP3 | chr2 | 119233751 | 119233752 | . |
| cg27214960 | CYP2E1 | -0.353830737 | 1.35E-13 | CYP2E1 | chr10 | 133529776 | 133529777 | . |
