# Supplementary figures and images for: A novel four-gene of iron metabolism-related and methylated for prognosis prediction of hepatocellular carcinoma
Source: Bioengineered. 2020 Dec 31;12(1):240–51. doi: 10.1080/21655979.2020.1866303 (PMC8806199; doi:10.1080/21655979.2020.1866303)

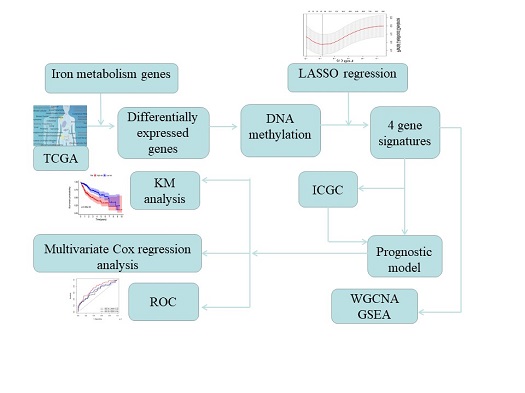

Supplement: Supplemental Material [file KBIE_A_1866303_SM2202.zip › supplement/Graphical Abstract.jpg]
